# Lived Experiences of Sexual and Gender Minorities in Solid Organ Transplantation: A Best-Fit Framework Synthesis and Inductive Thematic Analysis

**DOI:** 10.1177/20543581251331703

**Published:** 2025-05-29

**Authors:** Murdoch Leeies, Carmen Hrymak, David Collister, Emily Christie, Karen Doucette, Ogai Sherzoi, Tricia Carta, Ken Sutha, Cameron T. Whitley, Tzu-Hao Lee, Matthew J. Weiss, Sonny Dhanani, Julie Ho

**Affiliations:** 1 Gift of Life Program, Transplant Manitoba, Shared Health Manitoba, Winnipeg, Canada; 2 Section of Critical Care Medicine, Department of Medicine, Max Rady College of Medicine, University of Manitoba, Winnipeg, Canada; 3 Department of Emergency Medicine, Max Rady College of Medicine, University of Manitoba, Winnipeg, Canada; 4 Canadian Donation and Transplantation Research Program, Edmonton, AB, Canada; 5 Division of Nephrology, Department of Medicine, University of Alberta, Edmonton, Canada; 6 Division of Infectious Diseases, Department of Medicine, University of Alberta, Edmonton, Canada; 7 Centre for Healthcare Innovation, University of Manitoba, Winnipeg, Canada; 8 Division of Nephrology, Department of Pediatrics, School of Medicine, Stanford University, CA, USA; 9 Department of Sociology, Western Washington University, Bellingham, USA; 10 Section of Gastroenterology and Hepatology, Department of Medicine, Baylor College of Medicine, Houston, TX, USA; 11 Division of Abdominal Transplant, Department of Surgery, Baylor College of Medicine, Houston, TX, USA; 12 Transplant Québec, Montréal, Canada; 13 Division of Critical Care, Department of Pediatrics, Centre Mère-Enfant Soleil, CHU de Québec, Canada; 14 Department of Pediatrics, Division of Critical Care, Children’s Hospital of Eastern Ontario and University of Ottawa, ON, Canada; 15 Adult Kidney Program, Transplant Manitoba, Shared Health Manitoba, Winnipeg, Canada; 16 Section of Nephrology, Department of Internal Medicine, University of Manitoba, Winnipeg, Canada

**Keywords:** organ and tissue donation and transplantation, gender identity, sexual orientation, sexual and gender minorities, equity, safety, donor-derived infections, donor risk assessments, inclusive care

## Abstract

**Background:**

Organ and tissue donation and transplantation (OTDT) policies and practices lead to differential care for sexual and gender minorities (SGMs). The experiences of SGM patients and caregivers in the transplantation system have not been published. The perspectives of SGMs on how to best address existing inequities are not understood.

**Objective:**

To characterize the lived experiences of SGM patients and caregivers in solid-organ transplant health systems, as well as the perspectives and priorities of these individuals regarding SGM-relevant policies, practices and targets for system improvements.

**Methods:**

We conducted a series (N = 12) of one-on-one semi-structured interviews with a convenience sample of SGMs with lived experience of the OTDT system. We transcribed interviews verbatim and performed a formal qualitative analysis combining a best-fit framework synthesis and inductive thematic analysis.

**Results:**

We revealed novel targets for action to improve inclusive care in the transplantation system directly informed by the lived experiences of SGM patients and caregivers. Targets for improvement included (1) enhancements to shared decision-making between OTDT providers and patients, (2) transparent communication from OTDT organizations, (3) data-driven donor risk assessments, (4) expanded healthcare worker training, (5) inclusive physical care spaces, (6) recommendations for transgender and gender-diverse health system planning, (7) integrated sexual and reproductive healthcare services for transplant recipients, (8) increased SGM representation in medical education and care settings, (9) SGM and OTDT intersectional support networks, and (10) structural facilitation of SGM community advocacy efforts.

**Limitations:**

While thematic saturation was achieved with our sample, we recognize that not all SGM identities were represented. It remains likely that additional experiences, beliefs, and priorities exist in the SGM community.

**Conclusions:**

The emergent priorities and perspectives of SGMs with lived experience of transplant systems should inform patient-centered equitable health system advancements.

## Abrégé

**Contexte:**

Les politiques et pratiques actuelles entourant le don et la transplantation d’organes et de tissus (DTOT) entraînent des différences dans les soins des personnes membres de minorités sexuelles et de genre (MSG). Aucun article publié ne fait état des expériences des patients et soignants membres de MSG dans le système de transplantation. Le point de vue de ces personnes sur la meilleure façon de remédier à ces inégalités est encore mal connu.

**Objectifs:**

Caractériser les expériences vécues par les patients et soignants membres de MSG dans les systèmes de transplantation d’organes solides, et connaitre leur point de vue et leurs priorités en matière de politiques, de pratiques et de cibles d’amélioration du système pertinentes pour les MSG.

**Méthodologie:**

Nous avons mené une série d’interviews individuelles semi-structurées (n=12) auprès d’un échantillon de commodité composé de personnes membres de MSG ayant un vécu expérientiel du système de DTOT. Nous avons retranscrit textuellement le contenu des interviews et procédé à une analyze qualitative formelle combinant une synthèse du cadre le mieux adapté et une analyze thématique inductive.

**Résultats:**

Nous avons mis en évidence de nouvelles cibles d’action, reposant directement sur le vécu expérientiel des patients et soignants membres de MSG, pour favoriser l’inclusion dans les soins du système de transplantation. Ces cibles étaient les suivantes : 1) amélioration de la prise de décision partagée entre les prestataires de soins de DTOT et les patients; 2) communications transparentes des organismes de DTOT; 3) évaluations des risques pour les donneurs fondées sur les données probantes; 4) élargissement de la formation du personnel de santé; 5) espaces plus inclusifs pour les soins physiques; 6) recommandations pour la planification dans le système de santé pour les personnes transgenres et la diversité des genres; 7) services intégrés de santé sexuelle et reproductive pour les receveurs d’une greffe; 8) représentation accrue des MGS dans l’enseignement médical et les structures de soins; 9) réseaux de soutien croisés entre les MSG et les systèmes de DTOT; 10) facilitation structurelle des efforts pour la défense des intérêts des communautés des MSG.

**Limites:**

Bien que nous ayons atteint la saturation thématique avec notre échantillon, nous reconnaissons que toutes les identités des MSG n’étaient pas représentées. Il est possible que d’autres expériences, avis et priorités existent dans les différentes communautés des MSG.

**Conclusion:**

Le point de vue et les priorités émergentes des membres des MSG ayant un vécu expérientiel des systèmes de transplantation devraient guider les progrès des systèmes de santé plus équitables et centrés sur le patient.

## Background

Sexual and gender minority (SGM) populations face differential policies and practices in organ and tissue donation and transplantation (OTDT) systems compared to heterosexual and cisgender populations.^1^ SGMs, including Two-Spirit, Lesbian, Gay, Bisexual, Transgender, Queer, Questioning, Intersex and more identities (2SLGBTQI+) make up at least 10% of the global population with increasing proportions of people self-identifying as 2SLGBTQI+ in younger generations.^2^ SGMs receive a lower quality of healthcare with poorer outcomes than the general population.^3^ We have previously described OTDT system inequities, harms, and knowledge gaps relevant to SGMs.^4^ OTDT systems do not routinely collect relevant socioeconomic data to routinely quantify or monitor SGM-specific inequities.^5^ This leads to under-representation of SGM identities in observational research and health system planning.^6^ A lack of precision regarding self-identified gender vs. sex assigned at birth and under-representation in research make transgender and gender-diverse (TGD) patients particularly vulnerable to variable practice.^6^ Heterogenous regulations regarding SGM patients in OTDT systems between the United States and Canada expose an opportunity to update and standardize SGM-relevant policies and practices using contemporary evidence.^7^ We performed in-depth semi-structured interviews with members of an SGM OTDT research advisory team composed of SGM organ donors, transplant recipients and the caregivers of deceased organ donors. In this manuscript we describe SGM patient and caregiver experiences, perspectives and recommendations to improve the care of SGM patients in transplantation health systems. The experiences of SGMs in the organ and tissue donation (OTD) system are described separately.

## Methods

### Qualitative Methodology

We used best-fit framework synthesis and paired inductive thematic analysis methods to analyze interview transcripts.

### Participants, Recruitment, Sample Size, and Thematic Saturation

We recruited 12 participants representing living and registered donors, caregivers of deceased donors and transplant recipients from a pre-existing SGM OTDT research advisory team (Table 1). We performed semi-structured interviews with each. The University of Manitoba Research Ethics Board provided approval for this study (HS26020(H2023:172)).

**Table 1.** Participant Characteristics.

Participant no.	OTDT involvement	2SLGBQI+ identity
1	Registered donor	Gay, cisgender man
2	Deceased organ donor husband	Gay, cisgender man
3	Deceased organ donor caregiver	Ally, heterosexual cisgender woman, caregiver of a gay cisgender man who was a deceased potential donor
4	Deceased tissue donor husband	Gay, cisgender man
5	Deceased tissue donor caregiver	Ally, heterosexual cisgender woman, caregiver of a gay cisgender man who was a deceased potential donor
6	Living organ donor	Bisexual, cisgender woman
7	Transplant recipient	‘Queerly straight’, transgender man
8	Transplant recipient	Lesbian, cisgender woman
9	Transplant recipient	Gay, cisgender man
10	Transplant recipient	Gay, cisgender man
11	Transplant recipient	Gay, cisgender man
12	Transplant recipient	Gay, cisgender man

*Note.* 2SLGBTQI+ = Two-Spirit, Lesbian, Gay, Bisexual, Transgender, Queer, Intersex, and more.

We interviewed 12 out of 13 members of our advisory team as a convenience sample, with 1 member not being able to participate due to competing demands on their time. Thematic saturation was determined according to the Guest method which compares the proportion of new themes emerging from each interview to the total number of themes already identified.^8^ We did not pair participant identifiers with individual quotations to minimize the risk of participant identification.

### Procedure, Interview Guide Development, and Interview Schedule

We conducted one 60- to 90-min interview with each participant using a web-conferencing platform from July to August, 2023. Informed consent, including review of the interview guide, was obtained prior to each interview. Interviews were recorded verbatim and transcribed for analysis. Participants were remunerated at $50/h.

We took a semi-structured approach to our interviews with question domains informed by a scoping review of the extant literature on SGM identities in OTDT^1^ and input from advisory team partners and co-authors. Collaborators further defined specific questions and potential prompts. Interview domains included (1) benefits of SGM identities, (2) harms, (3) inequities, and (4) beliefs and opinions regarding specific policies, recommendations or identified gaps in care, and (5) descriptions of individual experiences within the OTDT system (Online Supplemental Appendix 1).

A single researcher (M.L.) with a pre-existing relationship with participants (through the advisory team) conducted each interview. This interviewer had detailed knowledge of the subject matter focus of the interviews which facilitated maximum extraction of useful information from participants.

### Data Analysis

Demographic data are summarized as counts.^8^ Transcripts were analyzed using Atlas.ti (version 22.1.0). We first charted findings using a best-fit framework synthesis structured according to interview domains (Online Supplemental Appendix 1). Interview domains were the initial structure onto which we applied charted findings. The selected domains were based on a scoping review on sexual orientation and gender identity in OTDT.^1^ This method has been described as transparent and pragmatic,^9^ facilitating the coding of initial data against an *a priori* defined framework. We paired an additional inductive thematic analysis to identify additional themes and concepts. We conducted our thematic analysis in a phased approach: becoming familiar with the data, generating initial codes, searching for themes, reviewing themes, defining, and naming themes and producing the report.^10^ We created a codebook to ensure reliability of coding. Two researchers (M.L. and C.H.) independently coded transcripts in duplicate. The generation of codes was iterative moving from open coding where codes are generated from the text, then through review and discussion codes were grouped and refined to identify themes that captured the essence of the primary data. Agreement on final codes was achieved by consensus.

### Positionality and Reflexivity

We are a diverse team of SGM and ally investigators, caregivers, and patient partners. In collaborating on this work, we shared an a priori intention to support health equity for SGM populations. We approached this project with the shared knowledge that harms and inequities exist for SGMs in OTDT^1^ and we defined our intention to further explore the experiences of SGM patients and caregivers from their own perspectives. We anticipated that novel themes and experiences not previously published may exist. We felt the unique familiarity with OTDT system issues developed among our patient and caregiver advisory team was a strength of our approach and necessary to explore their perspectives regarding health system policies and practices that are not transparently communicated or commonly known to patients.^1^ We anticipated deriving more meaning from interviews with these patients and caregivers who had reflected on how current policies and practices had impacted them, compared to those who may have experienced differential treatment but been unaware.

## Results

### Participants

Twelve people including caregivers of deceased organ donors (n = 3), registered and living organ donors (n = 2) and transplant recipients (n = 6) each completed a semi-structured interview lasting 60 to 90 minutes. Most participants self-identified as cisgender (n = 11) and gay (n = 7). Participant characteristics are summarized in Table 1.

### Thematic Analysis

Seven themes and related sub-themes emerged from the interview transcripts (Table 2), for the analysis focused on SGMs in transplant systems.

**Table 2.** Emergent Themes and Sub-Themes.

Theme	Sub-theme
Theme 1: Stigma, Discriminatory Criteria & Inertia to Change	1. Shared decision-making and risk tolerance 2. Impact of ‘increased infectious risk donor’ stigma
Theme 2: System & Structural Elements: Inclusive Care	1. Enhanced OTDT healthcare provider training 2. Institutional action 3. Trust in the OTDT health system 4. Physical spaces 5. Representation 6. Enhanced sociodemographic variable collection 7. Screening for HIV and viral hepatitis
Theme 3: OTDT Patient & Community Relations	1. Informed consent
Theme 4: Benefits, Strengths & Resilience of the SGM Community	1. Accessing transplantation 2. Sex-positive culture as a facilitator of sexual and reproductive health promotion
Theme 5: Transgender & Gender Diverse Considerations	1. Gender identity and health information systems 2. Material considerations 3. Opportunities to enhance care for TGD patients 4. Benefits of gender-affirming care
Theme 6: SGM Priorities, Harms & Opportunities for Improvement	1. Sex-positive sexual and reproductive healthcare 2. Knowledge and practice gaps 3. SGM and OTDT Community 4. Pediatric considerations
Theme 7: Intersecting Identities	1. Socioeconomic status and geography 2. Race, ethnicity, and migrant identities 3. Age 4. Ability

*Note.* OTDT = organ and tissue donation and transplantation; HIV = human immune deficiency virus; TGD = transgender and gender diverse; SGM = sexual and gender minorities.

### Theme 1—Stigma, Discriminatory Criteria, and Inertia to Change

#### Shared decision-making and risk tolerance

Current shared decision-making processes between transplant programs and transplant candidates were a target for improvement, specifically, enhanced understanding about the benefits and risks of accepting an increased infectious risk donor (IIRD) organ vs. a baseline risk organ. SGM transplant recipients expressed a favorable benefit-to-risk ratio in accepting organs from SGM donors, “because right now my trajectory is death. So I’d rather live. And, those just don’t seem like substantial risks . . .” (Table 3, Quotes 3.3.1.1-3.3.1.2).

**Table 3.** Theme 1—Stigma, Discriminatory Criteria, and Inertia to Change.

Sub-theme	Representative quotation(s)
3.3.1 Shared Decision Making & Risk Tolerance	3.3.1.1—“The problem comes in, is the education that patients are receiving when presented with kidneys that are at high risk. Simply saying a kidney is at high risk and expecting the patient to remember all the stuff that they got from their transplant evaluation is heading in the wrong direction already. . . if I’m accepting a high risk kidney, I need to know a little bit more than the fact that it’s a high risk kidney. I need to know, you know, hepatitis, what do we know, there are ways of correcting that now. I have lost count of how many people I know who are living with HIV and have like zero counts. These things need to be factored into the transplantation process.”
	3.3.1.2—“I felt really comfortable getting an organ from anyone in the queer community. I have some understanding of the risk of HIV and AIDS and hepatitis and what we can do post-transplant if those are potential issues. For me it was like I’d rather live and have some risk, right? Because right now my trajectory is death. So I’d rather live. And, those just don’t seem like that substantial risks in terms of all of this.”
3.3.2 Impact of ‘Increased Infectious Risk Donor’ Stigma	3.3.2.1—“So there was a conversation about high risk donors and that was during the transplant process. . . I remember at the time I was like wait like I’m also gay you know, and so that was kind of like a jarring thing to me. It was like, oh my gosh, like we’re considered high risk? Also, I was a child too, you know? I was not out to my pediatric team. Whenever you’re exposed to something like that as a child a kind of shapes the way of which you view your identity, so that was not really a great thing to experience.”

#### Impact of “increased infectious risk donor” stigma

Impacts of IIRD paradigms on SGM transplant recipients included shame for having their identities equated with disease transmission, uncertainty of the accuracy of donor risk assessments in TGD patients, and opportunities to enhance communication around the relevant risks and benefits of accepting an IIRD-labeled organ. Deficit-focused framing of SGM donors as being at increased risk of infections was particularly impactful to pediatric recipients with evolving sexual orientations (Table 3, Quote 3.3.2.1).

### Theme 2—System and Structural Elements: Inclusive Care

#### Enhanced OTDT healthcare provider training

Participants desired enhanced SGM expertise and cultural humility skills in their healthcare providers (Online Supplemental Appendix 2, Table 2, Quotes 3.4.1-3.4.1.4). “I think anybody who has contact with the patient . . . transport people getting you to a test, the x-ray technician, the phlebotomist . . . not just the nurses and the doctors. Everybody who’s in contact with the patient should be trained.” They wanted healthcare providers to mirror the language they used to describe themselves and their relationships, but not to make assumptions about their identities (Online Supplemental Appendix 2, Table 2, Quotes 3.4.1.5-3.4.1.6). Participants recommended framing the expansion of these skills as an opportunity to celebrate diverse patients’ identities. Participants cautioned against anchoring biases like the “trans broken arm” syndrome (Table 4, Quote 3.4.1.8).

**Table 4.** Theme 2—System and Structural Elements: Testing and Inclusive Care.

Sub-theme	Representative quotation(s)
3.4.1 Enhanced OTDT Healthcare Provider Training	3.4.1.1—“Better training you know in medical school and nephrology, you don’t get a lot of training on working with LGBTQ+ patients in general. And I’ve helped with some of the trainings. I’ve been a patient where people can ask questions. And I’m always amazed when I ask them how much training or interaction they’ve had with LGBTQ people. They’ll say things like, ‘oh, well we’ve read a chapter about LGBTQ people’. Or you know ‘we had someone come in and speak one day’ and it’s so minimal. A lot more of that training is needed. We have some antiquated policies around blood donation and organ donation that don’t make sense in terms of men having sex with men or sometimes trans men having sex with men are included in that, sometimes we’re not, you never know. Trans women are often included in that because you know, of the HIV and AIDS crisis, and the stigma around that being an LGBTQ+ disease.” 3.4.1.8 – “We often talk about the ‘trans broken arm syndrome’. So don’t assume everything we have is because we’re taking testosterone or taking estrogen or because we are trans people. We can break our leg and it’s not really testosterone or estrogen, or us being trans people. You know with kidney failure taking testosterone does increase creatinine a little, but it doesn’t impact kidney function in the way that some people think it would impact kidney function, so it’s a safe thing to be taking even if you are in kidney failure.”
3.4.2 Institutional Action	3.4.2.1—“I felt like my nephrologist was really good about it. He would be like ‘this is what the policy says I don’t necessarily agree with what the policy says, but I have to tell you what the policy is and what we have to abide by, but here’s what I think’. And so I think like you know, being able to communicate this and we are working on addressing equities in terms of policy. Instead of just being like, ‘okay, here’s the policy and because of who you are you can’t donate or you’re at any increased risk’.”
3.4.3 Trust in the OTDT Health System	3.4.3.1—“When I was going through the transplant process I was. . . I will be honest, I was very worried about compliance. So I was trying to do all the things I needed to do. I was reaching out to people outside of that group to ask questions about ‘what do we know about hormone use and kidney function? What do we know about trans people and transplantation? What do we know about these things?’ And so I didn’t ask my team a lot of things until after the transplants happened because I was worried about compliance stuff. Did we have some of those conversations? Yes, but I was very, I was careful in that process until after the transplant happened for sure.”

#### Institutional action

Participants felt institutions should support equitable care through transparent communication, acknowledgment of existing gaps, and recognition of successful equity initiatives (Table 4, Quote 3.4.2.1). Institutions could further support equitable care through creation and enforcement of non-discrimination policies, standardization of SGM-relevant OTDT policies and practices, and offering SGM-specific advocacy services. Novel descriptions of the need for intersectional support networks that included SGM identities along with lived experience of the OTDT system emerged (Online Supplemental Appendix 2, Table 2, Quotes 3.4.2.2-3.4.2.9).

#### Trust in the OTDT health system

SGM transplant recipients reported a particular need to appear “compliant” to access transplantation, at times hiding their own concerns or even identities (Table 4, Quote 3.4.3.1). Age and ethnicity further interacted in patient perceptions of needing to compromise their priorities to maintain a compliant reputation (Online Supplemental Appendix 2, Table 2, Quote 3.4.3.2). Some patients felt their very bodily autonomy was at stake, “as a transplant patient, you can feel that pressure even more because you are being evaluated based on being compliant. So if you are not compliant in something because you don’t want it to be done to your body. . . you constantly feel afraid.”

#### Physical spaces, representation, sociodemographic variable collection and screening for HIV and viral hepatitis

Additional sub-themes and representative quotations are presented in Online Supplemental Appendix 3.

### Theme 3—OTDT Patient and Community Relations

#### Informed consent

Opportunities to strengthen informed consent processes in OTDT were identified for transplant recipients, living donors and deceased donors. Transplant recipients related incomplete understanding of issues such as the risks and benefits of accepting IIRD organs, and pressure to consent to whatever was asked to ensure access to transplantation from health system gatekeepers. Living donors identified a lack of preparation for the realization that the recipient of their donation would be notified if any IIRD factors were identified. Voluntary disclosure of sensitive information by donors was conditional on what was specifically asked of them (Online Supplemental Appendix 2, Table 3, Quotes 3.5.1.1-3.5.1.4).

### Theme 4—Benefits, Strengths, and Resilience of the SGM Community

#### Accessing transplantation

SGM communities were framed as specific networks that transplant candidates could turn to in search of a living organ donor (Table 5, Quotes 3.6.1.1-3.6.1.4). Activism and self-advocacy were described as foundational components of SGM communities’ identities, developed through surviving shared cultural events like the HIV/AIDS epidemic. These strengths translated into advocacy in supporting transplant candidates through their OTDT journey (Online Supplemental Appendix 2, Table 4, Quotes 3.6.1.5-3.6.1.6).

**Table 5.** Theme 4—Benefits, Strengths, and Resilience of the SGM Community.

Sub-theme	Representative quotation(s)
3.6.1 Accessing Transplantation	3.6.1.1—“When I was diagnosed, I was really obviously upset with being diagnosed with end-stage kidney disease. I was really upset about what my timeline would look like, what my life would look like and my family was initially concerned about telling everyone and how people would think what people would think about me or any number of things. My queer family was completely the opposite where they were like ‘well we need to broadcast this, we need to find a donor, we need to figure out how we’re gonna pay for this’. Like it became like this is a community thing that we’re gonna do and you know a lot of people had said things like that because I did so much for the community and helped so many people that they wanted to see me succeed and that this was going to be a community effort. So an old friend set up a Go Fund Me account and started raising money so that when I could have a transplant we would have the money ready for that. And then you know, I put it out there. I said, ‘Hey everyone look, I have kidney failure. I’m going to need a transplant. Here’s a link to the site where you can get screened for potentially donating a kidney’. So I literally just put it out on Facebook. And I had over 70 people sign up who were willing to donate.”
	3.6.1.2—“Anything that expanded my social network I think was in a way beneficial when I was looking for a living donor. I ultimately ended up receiving a deceased donor transplant, but when I was trying to reach out to my networks, there were many people that came forward. Some of whom were through professional and other personal networks, some of whom were through networks of people that I knew through engagement with different queer groups and organizations.”
	3.6.1.3—“Yeah, I think that there can be an increased sense of community and shared experience within the queer community. There was somebody in the gay men’s chorus here that donated a liver to another member. . . I think it’s just in the queer community, recognizing the importance of community, and found family beyond just our shared experience. I think those are all important things that can drive people to become organ donors to people that they may not know or may not know well.”
	3.6.1.4—“I hope that I keep this kidney forever but if the day comes where it’s no longer viable, I hope that my community here would help me.”

#### Sex-positive culture as a facilitator of sexual and reproductive health promotion

A sex-positive culture in the SGM community was noted to be a strength, particularly in supporting prevention, detection, and treatment of sexually transmitted infections (Online Supplemental Appendix 2, Table 4, Quote 3.6.2.1).

### Theme 5—TGD Considerations

#### Gender identity and health information systems

Universal asking about pronouns and gender identity was an example of an affirming practice (Table 6). Most transplant recipients said they had never been asked to confirm their gender identities. “No! Out of the 2 transplants that I’ve had, it’s never been a discussion.” Describing the options for sex assigned at birth vs. gender identity when registering to be a deceased organ donor, one interviewee noted the binary options available “Absolutely, M or F. Just one or the other.” Another participant noted positive changes at their current transplant center where confirmation of pronouns and gender identity have become a routine part of the clinical encounter (Online Supplemental Appendix 2, Table 5, Quotes 3.7.1.1-3.7.1.4).

**Table 6.** Theme 5—Transgender and Gender-Diverse Considerations.

Sub-theme	Representative quotation(s)
3.7.1 Socio-demographics	3.7.1.3—“I think improvements could look like. . . being a space that feels a little bit more welcoming and open to people of all different types of sexual identity and gender identity. I also believe that asking people about their sexual identity and gender identity in a questionnaire or in person would be a great step.”
3.7.2 Physical Care Spaces	3.7.2.1—“I also had a kidney biopsy done at the local hospital, and that was a really horrible experience. . . So they put me on a floor with other men, fine. I had to share a room with another guy. Sharing the room was not comfy in general and as a trans person is really not comfy. And then, the problem that occurred is that all of the equipment that they brought me to urinate was like one of those things that. . . I was like ‘I can’t use this’ and they were like, ‘well. . . you just, you have to use it’. And I was like, ‘well I can’t, this isn’t gonna work’. And they were like, ‘well, this is what we have for you’. And I was like. . .’what?’. But the weirdest part is I was on this floor with men, they were treating me like other men, even though I didn’t necessarily have exactly the equipment and then they were still messing up my pronouns.”
3.7.3 Opportunities to Enhance Care for Transgender Patients	3.7.3.2—“I feel like it’s often presented as like ‘we don’t have the expertise, we wanna make sure you get the right care’, when behind the scenes it’s really ‘I don’t necessarily feel comfortable with you’. And we as trans people, we know that. . . we see it. We see you when you say that, right? Because what you would do if you felt like you didn’t have the expertise or you didn’t have the knowledge base? You would actually find someone and refer us to that person and help us on that journey instead of just putting your hands up in the air and saying, ‘look I can’t help you’. The majority of us as trans people, we know what you’re doing when you say ‘hey, I can’t help because I’m not an expert’ and you don’t.”
	3.7.3.4—“When I started the process every time I would go to the doctor I’d have to do this long explanation about being trans and what that was like and I just. . . I almost was like, I just wanna die. Just let me die because this is too much to have to like have to explain my identity and go through this.”
	3.7.3.5—“I remember having a conversation with the Transplant Coordinator. And I was like look, I don’t know that this (renal function) calculation is correct and we need to have more conversation about this calculation. And she said to me, “it sounds like you’re really trying to get an organ transplant and when you get an organ transplant, this is a lifelong thing and you’re gonna have to take care of it and you’re not gonna want one sooner than you need one”. And it was so dismissive and she and her last comment was “and we don’t even know if you’ll be able to get one”. I felt like I was often trying to ask questions that they didn’t think a patient should be asking or telling them things that they didn’t think a patient should be telling them.”
	3.7.3.6—“I have been out and what I think has been hard to see is the number of trans people around the world who have contacted me about their own issues with chronic kidney disease and feeling that they haven’t been heard. They haven’t been seen, that they’ve been taken off testosterone, taken off estrogen asked to de-transition. They’ve been told that they couldn’t have a transplant unless they de-transition, told that they could never transition if they do get a transplant. So the number of people who are experiencing those issues and have limited access or no access to quality affirming health care is really upsetting.”
	3.7.3.8—“There was no conversation around the risk and benefits in terms of stopping. The Nephrologist communicated back to my doctor at the health center that this was her recommendation. So my doctor wouldn’t prescribe any more hormones. So I was cut off.”
	3.7.3.9—“When I was in a rural community and accessing rural healthcare, it was horrible. It was so bad that I felt like in some ways I’d rather die than go through this process of being, feeling humiliated and not exactly ridiculed but having that. . .overhead. My wife and I decided that we would have to live reasonably close to a major metropolitan area and only seek health care from a major university centre. We also decided that we can’t live in a conservative or red state. When we were on the job market, we had opportunities in Texas, we had opportunities in Florida and that was no-go. That was an absolute no-go because of my health and my trans status, those are not safe destinations for us.”
	3.7.3.16—“It was a repeated challenge. I think the hard part is that most places have a designated kind of cut-off for being listed and what I understood at the time is it was hard to overwrite the system. Your cut-off is based on how your bloodwork is entered. By the time I had port placement for dialysis, I was down to I think it was in 8 for kidney function on a male scale. And I was down to I think 91 pounds at the time. It was pretty rough and when I had the port placement I had a significant bleed, almost died in that process. They had to do emergency dialysis, I was in the hospital for several days. That was a pretty tense process. It was unclear because there was no literature about what exactly to do and there still isn’t a lot of literature. We’re still trying to develop that, right? It was unclear based on my body type, the sex organs I do have, the hormone levels. . . all the things we needed to figure out and factor into this equation. We waited as long as possible to do dialysis and I think we both recognize that we waited too long.”
	3.7.3.17—“Now we know because of muscle mass and all these other factors, we know that that’s really problematic, especially because in all my documentation I’m coded as male. . . on my birth certificate, driver’s license, everything is coded male. So everything is run as male, but if you take away my hormones, then that doesn’t that doesn’t work anymore. So it’s really a problematic piece where then, I could do these calculations and know I’m not gonna be put on the transplant list when I need to be put on the transplant list because you’re calculating my numbers wrong. And it was a very scary time in terms of figuring out like how do I. . . how do I communicate this to my doctors? And I’m not a doctor, right? Like how do I tell them this and get them to listen because they’re not really listening?”

#### Material considerations

Interviewees identified practical considerations like available urine-collection systems and gender cohorting on hospital wards as factors that may uniquely impact a transgender patient’s experience (Table 6, Quote 3.7.2.1). Reflecting on their hospital stay in the peri-transplant period, one recipient recounted “I don’t think there are gender-neutral bathrooms. I remember the way the floor was divided, of girls’ rooms and boys’ rooms. And I also think that in terms of the activities in the hospital, it was also a boy versus girl type of thing. So I would understand that for someone that’s gender queer or someone that is non-binary that might be an issue for them.”

#### Opportunities to enhance care for TGD patients

Participants identified an ongoing need for enhanced gender-affirming care in OTDT systems. They identified under-recognized barriers for transgender people accessing care, for example, structural inequities such as a lack of expertise and education among healthcare providers leading to a void in the healthcare network. TGD patients expressed feelings of abandonment when lack of expertise was used as rationale to decline engaging in their care. When TGD patients were able to access care, they described harms related to the additional burden of needing to educate the healthcare team about their identity and specific needs (Table 6, Quotes 3.7.3.2 and 3.7.3.4) (Online Supplemental Appendix 2, Table 5, Quotes 3.7.3.1-3.7.3.4).

Participants highlighted existing knowledge gaps like the application of sex-based biometrics in TGD patients and described a disproportionate burden of having to research their own health issues and advocate for personalized care plans (Table 6, Quote 3.7.3.5). Transgender patients having their gender-affirming hormone therapy (GAHT) discontinued or withheld without their input to access transplantation was a recurrent described harm (Table 6, Quote 3.7.3.6) (Online Supplemental Appendix 2, Table 5, Quotes 3.7.3.6-3.7.3.7). Participants framed this lack of shared decision-making in stopping GAHT as a violation of patient autonomy (Table 6, Quote 3.7.3.8).

Less access to gender-affirming care was reported in smaller and rural centers with TGD transplant recipients describing how their career choices and housing selection were restricted by the need to access inclusive medical care (Table 6, Quote 3.7.3.9). Patients reported needing healthcare directives to avoid certain facilities in the event they could not advocate for themselves due to distrust that they would receive acceptable care. Travel for personal or professional reasons was also limited by the need to have access to inclusive emergency care (Online Supplemental Appendix 2, Table 5, Quotes 3.7.3.10-3.7.3.11). Patients described incidents where their identities were directly invalidated by healthcare staff, exposing a need for enhanced healthcare worker training (Online Supplemental Appendix 2, Table 5, Quotes 3.7.3.12-3.7.3.13).

Reported transgender-specific knowledge gaps in OTDT included invisibility and under-representation of transgender identities in research, uncertainty in how sex-based biometrics should be applied to transgender patients on and off GAHT, and incomplete understanding of the risks and benefits of GAHT in the peri- and post-transplant period. Transgender-specific knowledge gaps directly impacted access to pre-emptive kidney transplantation due to kidney function calculations modified by sex assigned at birth rather than gender identity (Online Supplemental Appendix 2, Table 5, Quotes 3.7.3.14-3.7.3.15). Interviewees described other complications of organ failure due to barriers in accessing pre-emptive transplantation including complications from a dialysis catheter placement which could have been prevented had their listing for transplant not been delayed due to sex-based kidney function criteria. Additional stress in needing to self-advocate and feeling unheard by OTDT providers was described as a harm experienced by transgender patients (Table 6, Quotes 3.7.3.16-3.7.3.17) (Online Supplemental Appendix 2, Table 5, Quotes 3.7.3.16-3.7.3.19).

#### Benefits of gender-affirming care

Participants shared their hopes for equitable care in the future and more personalization of care for transgender people. Examples of affirming experiences included the structural integration of a patient navigator/advocacy service (Online Supplemental Appendix 2, Table 5, Quote 3.7.4.1). Patients described relief and validation when they were able to access inclusive gender-affirming care. “At my current transplant institute they immediately put me back on hormones. I remember the first time talking to a transplant doctor. I was like, ‘hey, so look here’s the deal, I’m a transgender guy I’ve had hormones for almost 20 years. Here’s what’s happening.’ And she was like ‘okay.’ And I was like, ‘wait, do like, did you hear me? Like I am a trans guy. And so I may need different care.’ She was like ‘okay,’ like it was like nothing. And that was repeatedly my experience at my current hospital. Which was wonderful. It’s absolutely wonderful.”

### Theme 6—SGM Priorities, Harms and Opportunities for Improvement

#### Sex-positive sexual and reproductive healthcare

Participants reported insufficient sexual and reproductive healthcare in their transplant programs. Some attributed this to vulnerabilities in the unique intersection of care they inhabit between primary healthcare and subspecialty transplant care (Table 7, Quotes 3.8.1.2-3.8.1.9). SGM-identifying transplant recipients revealed several barriers to accessing sex-positive care. Healthcare providers framing sexual health interventions as optional, unnecessary, or separate from more important medical issues was a barrier to holistic care. Access to pre-exposure prophylaxis (PrEP) and M-Pox vaccination were specific examples of interventions framed as unnecessary (Online Supplemental Appendix 2, Table 6, Quotes 3.8.1.10-3.8.1.11).

**Table 7.** Theme 6—SGM Priorities and Opportunities for Improvement.

Sub-theme	Representative quotation(s)
3.8.1 Sex-Positive Sexual & Reproductive Healthcare	3.8.1.2—“I am Gen X and so I’ve been dealing with kidney disease now for almost 20 some years, and since my first introduction to kidney disease sexuality was not discussed at all. When I went in for my transplant evaluation, sexuality was not talked about.”
	3.8.1.3—“I found that none of my physicians asked about my sexuality. None of them, none of them ever engaged in my sexuality, and these are my transplant teams. These are my nephrology nurses and doctors and the transplant evaluations team. None of them.”
	3.8.1.4—“There just isn’t much. . . that’s dealing with LGBTQ issues as it relates to kidney health. But understanding that people who sit in the dialysis chair, people who get transplants also have a sexual identity. In fact, sexual identity is usually not even discussed for the most part.”
	3.8.1.5—“Now that I’m thinking about it, I don’t think my team really did talk to me about my sexual health. And being immunocompromised, I don’t think they asked about what kind of sex I was having, what kind of protection I was using.”
	3.8.1.6—“There was none. Not just about sexual orientation or gender identification. . . There’s no talk from any team that I’ve ever had about anything, slightly sexually oriented. It has just been completely omitted from the conversation.”
	3.8.1.7—“They’ll talk about impacts of Prednisone on weight. And you know how you’re gonna have to take it and why you needed it. There they talk about CellCept® and technical impacts on the body. There is no conversation about how as an immunosuppressed person that puts you at higher risk for transmission of sexually transmitted diseases. You have to be more careful about who you select as your partner. None”
	3.8.1.8—“No, that’s never been explicitly brought up. I’ve had a little bit of discussion about that with my primary care doctor, but again, no real directed discussion about sexual health or sexual practices, risk of different activities, anything like that.”
	3.8.1.9—“There was not like a whole presentation or giving me education or anything. . . sexual education for transplant patients who are gay? There’s nothing like that. Nope, never. Done my own education.”
	3.8.1.16 -”I have felt that I have to drive a lot of those things and I’m very fortunate that I have a lot of knowledge in this area and I have the privilege and expertise to be able to navigate the limited amount of literature in this in the space, but that’s definitely not the case for the majority of people.”
	3.8.1.17—“I think a lot of times still though, it doesn’t come up explicitly unless I bring it up. And that puts a lot of the onus on the patient to kind of talk about what their concerns are about. And sometimes to educate providers about different kinds of concerns that are specific to gay men. For example things like PrEP use, different considerations that we as transplant patients might need to have around different kinds of viral STIs even. Having a transplant, we’re at risk for more complications for different kinds of infections.”
	3.8.1.18—“In looking the through literature, there’s not really great guidance for the screening of HPV driven malignancies in men who have sex with men and with transplants in particular. There’s some guidance for initial screening in the general men who have sex with men population. There’s guidance for transplant patients with uteruses to look for cervical cancer. But again, there’s this intersection where there’s maybe not data to drive guidance. I felt like I was in a no man’s land of trying to figure out what the follow-up was gonna be and discussing this with my care providers.”
	3.8.1.19—“There was this thing with the monkey pox vaccine where it was viewed as unnecessary. (My team) didn’t really think it would be very useful and I kind of really had to push it to say it is something I really do need to be aware of because I had contact with someone that had monkey pox and it was so so scary. I was like, ‘this is insane, like I need to get the vaccine ASAP’. And my care team was like, ‘why?’. And I told them I had contact and so it was just this very weird dilemma where I was immunocompromised and they would be so scared of like a tiny speck of dust but they didn’t care about the monkey pox vaccine. And so I did have to push it.”
	3.8.1.20—“I don’t think (trans-specific issues) were something that my team necessarily brought up or highlighted. There’s kind of a standard question I ask every time I have to have a procedure or anything like “how will you take care of me as a trans patient?”. And so whenever we’re doing pre-op anything or whatever, that’s kind of my standard question. Instead of someone telling me how they’re gonna take care of me as a trans patient.”
3.8.2 Knowledge & Practice Gaps	3.8.2.2—“It’s difficult for transplant providers, as being very subspecialty, to necessarily become the experts or be the ones to do be doing everything, that may be better suited under primary care, but I think there are specific intersections where for specific issues there could be a role. For example, like I mentioned these risks for these different virally mediated STIs that transplant patients may have more adverse outcomes from. HPV like I mentioned, HSV etc. Being well-versed in things like PrEP, because there’s the potential for interactions with medications, so knowing about that and bringing that up with patients. Not just sexual orientation minorities, but everybody, to be able to have that discussion with all sexually active individuals and being able to offer that and know when that would be potentially beneficial for different people. I think it’s important. I think especially for me, I never had conversations about PrEP use with my transplant doctors, but it was a huge area of concern for me because of a potential nephrotoxicity of Truvada in particular and trying to weigh the risks and benefits of PrEP use.”
3.8.3 SGM & OTDT Community	3.8.3.1—“As a gay man with a with a kidney transplant, I’ve always sought out community of others with similar lived experiences. I felt like when I was in gay spaces I rarely ran into other people with transplants that were able to identify with similar experiences that I went through, or even with any kind of chronic illness. Many times images we see in the media of what it is to be a gay man out in the world, like at some circuit party or something, are very image driven and this quote unquote like picture of health kind of thing. I think this has roots in the HIV AIDS epidemic. But I never saw myself represented, whether it be my chronic illness or oftentimes being an Asian American, I think I am oftentimes excluded from portrayals that are seen in the media. So at the same time, on the transplant side, even back to the time when I was first diagnosed with kidney disease, when I knew that I needed to be a get a transplant, I sought out people that were going through similar experiences. Being a young adult dealing with waiting for a kidney transplant, it was difficult for me to meet other people that had similar experiences. Most of the people that I met when I was in clinic tended to be quite a bit older and didn’t have a similar kind of stage to what I had. Eventually I found community in different volunteer organizations and Facebook groups, online forums. But again, in those places, I didn’t meet other people that identified with my queer identity there, and at times encountered outright hostility. For example, when posting information about why it was important to have gender affirming care, intersections with transplant care or kidney care or things about Pride Month, it was met with outright homophobia and homophobic comments in those Facebook forums.”
3.8.4 Pediatric Considerations	3.8.4.1—“The thing about sexual orientation is that it can’t be ignored, right? You can’t hide it and I was scared if I told my care team they would discriminate against me. That added a real sense of fear growing up. Just growing up gay is hard. But growing up as a transplant recipient gay—can you imagine that? It’s really difficult. I felt this like extreme low sense of self-esteem. Adding all these identities onto me . . . like disabled and person of color and queer on top of that . . . It just felt kind of overwhelming and I had such low self-esteem and then that kind of like impacted the first time I had sex because in all honesty the first time I had sex was sexual assault. I was essentially talked to like this random stranger online and then I kind of like led myself down this road where I got taken advantage of and essentially raped by a random stranger.” 3.8.4.2—“Being a pediatric queer patient has its own caveats because I would like to just emphasize—you don’t even know who you are yet and you’re hit with this big thing. This tremendous amount of pain that you experienced at such a young age, and then being able to process that trauma, and then having the queer identity on top of it which is already stigmatized . . . So you have to learn how to exist in this world as you are at such a young age and it’s really really hard. Overall, that affects us. It’s a little bit different than someone that had a transplant when they were older. We have a different set of experiences. We are similar, but also the pediatric transplant person’s experiences establishing their identity, establishing who they are. . . And identity is so important the queer experience. And identity is so important to the transplant experience. There are these things for the pediatric transplant recipient, that just might be a little bit different than someone with an adult transplant recipient experience.”

Insufficient healthcare provider training in sexual and reproductive health for SGM populations was a noted barrier. “What I discovered is that it wasn’t so much my sexuality that they didn’t want to talk about. They didn’t know how to talk about sex, period. I’ve never seen my nephrologist blush as much as when I asked him about sexual function and kidney disease, and how I would like to be sexually active. And I swear he turned multiple shades of red right in front of me. It was like, ‘okay, I’m asking you this question but it’s obvious it’s not my sexuality that you’re nervous about. . . it’s that you don’t know how to talk about sex.” Ingrained heteronormative assumptions can lead to misunderstandings when taking a sexual history and SGM patients may be uncomfortable disclosing sexual health concerns, further supporting the need for training healthcare providers in inclusive sex-positive care (Online Supplemental Appendix 2, Table 6, Quotes 3.8.1.13-3.8.1.15). Many transplant recipients described a disproportionate need to research their own health issues and self-advocate for access to sexual and reproductive healthcare (Table 7, Quotes 3.8.1.16-3.8.1.20).

Described sexual and reproductive health-related harms included stigmatization of SGM sex as being inherently “risky,” being prescribed abstinence over other risk-reductive strategies, transgender patients being forced to de-transition or denied GAHT to access transplantation, perceptions of SGM patients being used for medical teaching, and having one’s identity disrespected or disregarded (Online Supplemental Appendix 2, Table 6, Quotes 3.8.1.21-3.8.1.24).

#### Knowledge and practice gaps

Interviewees identified a unique need for enhanced sex-positive sexual and reproductive healthcare as a gap that transplant recipients were specifically impacted by. Transplant visits were identified as opportunities to interface with the medical system and participate in sexual and reproductive health promotion. One transplant recipient shared the disproportionate amount of time and advocacy it took to be able to access human papillomavirus (HPV) vaccination recognizing internal and external barriers (Table 7, Quote 3.8.2.2-3.8.2.3). Interviewees identified additional opportunities to integrate preventative health measures into the transplant system through enhanced education and screening for virally mediated malignancies for all patients. Coordination of care between primary care and subspecialty transplant care as a barrier to be overcome was a repeated theme (Online Supplemental Appendix 2, Table 6, Quotes 3.8.2.1-3.8.2.5).

#### SGM and OTDT community

Participants described the development of spaces where SGM transplant recipients could meet to share information and provide peer support in reaction to queerphobic discrimination they had faced in general transplant support networks. Representation of the diversity of SGM people including transplant recipients, people with disabilities, different races and ethnicities was described as a benefit of SGM-inclusive transplant support networks (Table 7, Quote 3.8.3.1). Virtual spaces like online discussion groups were used by SGM transplant patients in search of inclusive peer support. Interviewees also noted the potential of an SGM OTDT network to help SGM transplant candidates find a living donor (Online Supplemental Appendix 2, Table 6, Quotes 3.8.3.1-3.8.3.9).

#### Pediatric considerations

Pediatric transplant recipients were felt to be uniquely vulnerable to harms in the context of evolving identities. The interplay of developing sexual identities and sexual orientations along with a medicalized identity, a disability identity, ethnicity, and migrant status uniquely impacted patients. Participants called for more awareness of the complexities of supporting SGM pediatric patients with organ failure and transplantation. Described harms of non-inclusive care included low self-worth, isolation, and exposure to sexual violence (Table 7, Quotes 3.8.4.1-3.8.4.2).

### Theme 7—Intersecting Identities

Interviewees highlighted how additional intersecting identities may uniquely impact an SGM patient or caregiver’s journey through the OTDT system. (Online Supplemental Appendix 4).

### Improving SGM Care in OTDT

**Figure 1.**
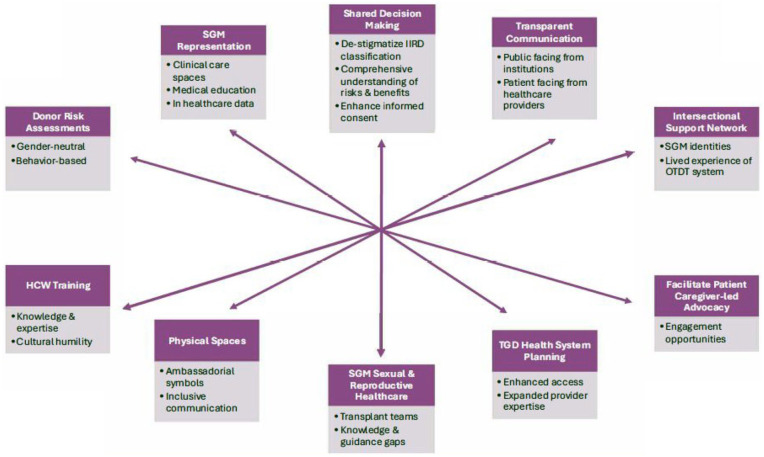
Emerging targets for action. *Note.* IIRD = increased infectious risk donor; SGM = sexual and gender minorities; OTDT = organ and tissue donation and transplantation; TGD = transgender and gender diverse; HCW = healthcare worker; NAAT = nucleic acid amplification test.

Targets for system improvement extracted from patient and caregiver interviews are summarized in Figure 1.

## Discussion

### TGD Considerations

Representation of TGD identities and experiences in OTDT systems is lacking in published literature.^1^ Participant descriptions of delaying or avoiding needed healthcare due to fears of discrimination were poignant and are consistent with barriers to accessing healthcare for TGD populations in the larger health system.^11-15^ Transgender transplant recipients described a lack of trust in the OTDT system due to experiences of discrimination and threatened access to GAHT. Described forced de-transitioning or barriers to accessing GAHT in exchange for access to transplantation is a critical finding that warrants a coordinated position statement from decision-making bodies in OTDT. Just as transplantation is life-saving in end-organ failure, GAHT is life-saving in TGD patients with gender dysphoria.^16^ Physicians are the gatekeepers of medical and surgical transition therapies which are essential to living as one’s true self for many TGD people. Decisions to withhold GAHT should be evidence-based and include shared decision-making with patients. There are significant knowledge gaps in OTDT for TGD patients which need to be addressed to support inclusive data-driven care.^1,16-18^ Interpreting sex-based biometrics in TGD patients and the potential effects and interactions of GAHT in post-transplant patients are priority areas of research.^16-18^ Interviewees clearly identified the need for enhanced healthcare worker training in TGD health and cultural humility. ODOs in Canada have also self-identified gaps in training in the provision of SGM-specific care.^5^

Trans-joy was evident through participant descriptions of examples of inclusive and affirming care. Medical literature on TGD experiences is often from a deficit-based perspective to describe inequities^15^ but strengths-based approaches can identify and reinforce human capacities rather than deficiencies.^19^ The framing of a marginalized population in medical literature permeates both education and clinical practice. A strengths-based approach offers an opportunity to focus on wellness and thriving and to de-stigmatize deficit narratives that can subconsciously influence social perspectives and perpetuate deficit-based healthcare approaches.^19-22^ Recognizing TGD identities through inclusive collection of gender, pronouns and other sociodemographic data, acknowledging the importance of GAHT and facilitating access where appropriate, exhibiting an understanding of TGD specific issues and willingness to provide ongoing care without threatening this identity were all examples of clinicians supporting trans-joy in our interviews.

### Sexual and Reproductive Healthcare

Transplant recipients face a unique barrier in accessing sexual and reproductive healthcare, which is typically provided in primary healthcare settings, as they receive much of their care in specialty transplant spaces. They described a deficit in this expertise in transplant providers, yet primary care teams were at times uncomfortable providing this care due to a lack of expertise in transplant recipient-specific issues. SGM transplant recipient interviewees described their transplant teams as being ill-equipped to provide sexual and reproductive health care, a finding that is echoed in the general transplant recipient population.^23-26^ Transplant providers themselves also identify a need for enhanced training.^25,23,27,28^ Team-based models that integrate primary care providers into the specialty care of transplant recipients might address this gap in care. Recommendations to promote sexual and reproductive health for general populations of transplant recipients have been published^29,30^ but specific guidance for SGM populations is lacking. Evidence to inform post-transplant fertility along with GAHT is needed.

Transplant recipients have a significantly increased risk of HPV-associated malignancies, treatment refractory HPV disease, and decreased immune response to HPV vaccination compared to the general population.^30^ Guidance on screening, vaccination, and confirmation of immunity for HPV in transplant recipients exist but uptake and implementation of these recommendations may be heterogenous, as was the experience of some interviewees.^30^

Our participants reported barriers in accessing HIV preventative PrEP which is highly effective at preventing sexual transmission of HIV.^31,32^ Awareness of potential drug interactions in transplant recipients (primarily with calcineurin inhibitors) and education on PrEP prescribing and monitoring could increase access to these therapies that have revolutionized HIV prevention efforts.^29,33^ Additional questions about the efficacy and safety of HIV post-exposure prophylaxis for transplant recipients being offered increased infectious risk donor organs or tissues are unanswered. Concerningly, some participants reported healthcare providers recommending abstinence to prevent HIV, which has been shown to be ineffective.^34^ While the use of HIV PrEP is highly effective at preventing HIV transmission through sexual contact,^31,32^ there is limited experience in the peri-transplant setting with concerns about the risk of renal impairment.

Interviewees described a disproportionate burden of self-advocacy with multiple providers for access to SGM-specific sexual and reproductive healthcare. Decreasing access barriers and supporting shared decision-making with patients is needed. Descriptions of pediatric transplant candidate and recipient perspectives identified a unique intersection where the co-evolution of sexual identity, orientation, and gender identity in combination with a chronic illness made these patients uniquely vulnerable to stressors and at risk of harms including sexual violence when communication was not open and transparent. Patient and caregiver perspectives combined with healthcare provider self-assessments all support that comprehensive education for staff in all parts of the OTDT system on SGM health and cultural humility is needed.

Recognizing the need for enhanced programming, interviewees described SGM populations as having enhanced capacities in social and medical activism born out of a history of discrimination in these spaces. They identified these skills as supporting a sex-positive culture that was uniquely able to engage in HIV detection, prevention, and treatment efforts. In Canada, the proportion of new HIV infections in SGM populations is decreasing and SGM populations are more likely to be aware of their status, engage in regular testing, use effective HIV prevention methods, and for those living with HIV more likely to have undetectable viral levels.^35^ These skills make SGM populations ideal partners in enhancing sexual and reproductive healthcare.

### Strengths and Limitations

This study characterizes novel lived experiences of SGM patients and caregivers in transplantation systems. These have not been previously published in the medical literature. Patients and caregivers identified actionable targets to enhance inclusive care. We recognize that not all SGM identities or perspectives have been represented, in particular TGD, bisexual and lesbian perspectives were minimally represented and this is a limitation of our sample. For transformational health system change to occur, patient and caregiver experiences will need to be combined with the perspectives of healthcare providers and OTDT system stakeholders.

## Conclusions

Our study characterized the priorities and perspectives of SGM patients and caregivers with lived experience of the organ transplantation system. Actionable targets include addressing knowledge and policy gaps for TGD patients and processes to ensure support and safety for SGM pediatric patients. Patients and caregivers called for enhanced healthcare provider training in SGM-specific topics. Expanded sexual and reproductive health service offerings were desired by transplant recipients who described barriers in accessing this care. OTDT systems should use these patient and caregiver priorities as catalysts to further improve inclusive healthcare delivery.

## Supplemental Material

sj-docx-1-cjk-10.1177_20543581251331703 – Supplemental material for Lived Experiences of Sexual and Gender Minorities in Solid Organ Transplantation: A Best-Fit Framework Synthesis and Inductive Thematic Analysis Supplemental material, sj-docx-1-cjk-10.1177_20543581251331703 for Lived Experiences of Sexual and Gender Minorities in Solid Organ Transplantation: A Best-Fit Framework Synthesis and Inductive Thematic Analysis by Murdoch Leeies, Carmen Hrymak, David Collister, Emily Christie, Karen Doucette, Ogai Sherzoi, Tricia Carta, Ken Sutha, Cameron T. Whitley, Tzu-Hao Lee, Matthew J. Weiss, Sonny Dhanani and Julie Ho in Canadian Journal of Kidney Health and Disease

sj-docx-2-cjk-10.1177_20543581251331703 – Supplemental material for Lived Experiences of Sexual and Gender Minorities in Solid Organ Transplantation: A Best-Fit Framework Synthesis and Inductive Thematic Analysis Supplemental material, sj-docx-2-cjk-10.1177_20543581251331703 for Lived Experiences of Sexual and Gender Minorities in Solid Organ Transplantation: A Best-Fit Framework Synthesis and Inductive Thematic Analysis by Murdoch Leeies, Carmen Hrymak, David Collister, Emily Christie, Karen Doucette, Ogai Sherzoi, Tricia Carta, Ken Sutha, Cameron T. Whitley, Tzu-Hao Lee, Matthew J. Weiss, Sonny Dhanani and Julie Ho in Canadian Journal of Kidney Health and Disease

sj-docx-3-cjk-10.1177_20543581251331703 – Supplemental material for Lived Experiences of Sexual and Gender Minorities in Solid Organ Transplantation: A Best-Fit Framework Synthesis and Inductive Thematic Analysis Supplemental material, sj-docx-3-cjk-10.1177_20543581251331703 for Lived Experiences of Sexual and Gender Minorities in Solid Organ Transplantation: A Best-Fit Framework Synthesis and Inductive Thematic Analysis by Murdoch Leeies, Carmen Hrymak, David Collister, Emily Christie, Karen Doucette, Ogai Sherzoi, Tricia Carta, Ken Sutha, Cameron T. Whitley, Tzu-Hao Lee, Matthew J. Weiss, Sonny Dhanani and Julie Ho in Canadian Journal of Kidney Health and Disease

sj-docx-4-cjk-10.1177_20543581251331703 – Supplemental material for Lived Experiences of Sexual and Gender Minorities in Solid Organ Transplantation: A Best-Fit Framework Synthesis and Inductive Thematic Analysis Supplemental material, sj-docx-4-cjk-10.1177_20543581251331703 for Lived Experiences of Sexual and Gender Minorities in Solid Organ Transplantation: A Best-Fit Framework Synthesis and Inductive Thematic Analysis by Murdoch Leeies, Carmen Hrymak, David Collister, Emily Christie, Karen Doucette, Ogai Sherzoi, Tricia Carta, Ken Sutha, Cameron T. Whitley, Tzu-Hao Lee, Matthew J. Weiss, Sonny Dhanani and Julie Ho in Canadian Journal of Kidney Health and Disease
