# Supplementary material for: Lived Experiences of Sexual and Gender Minorities in Solid Organ Transplantation: A Best-Fit Framework Synthesis and Inductive Thematic Analysis
Source: Can J Kidney Health Dis. 2025 May 29;12:20543581251331703. doi: 10.1177/20543581251331703 (PMC12126676; doi:10.1177/20543581251331703)
Supplement: sj-docx-1-cjk-10.1177_20543581251331703 – Supplemental material for Lived Experiences of Sexual and Gender Minorities in Solid Organ Transplantation: A Best-Fit Framework Synthesis and Inductive Thematic Analysis [file sj-docx-1-cjk-10.1177_20543581251331703.docx]

**Characterizing Lived Experiences of Sexual and Gender Minorities in Organ and Tissue Donation and Transplantation: A Qualitative Analysis**

*Interview Guide*

*Introductions, Sociodemographics, Rapport Building, Personal Narratives*

I’d like to start by asking you some general getting-to-know-you questions. These are standard questions that we’re asking everyone, some answers might seem obvious to you, but we ask you to still answer to the best of your ability.

- - How would you like us to address you?
    - (prompt: Name, any honorifics (Mr., Ms., Mx. By name))
  - What are your pronouns?
    - (prompt: She/her, he/him, they/them, ze/zir, a different choice)
  - What is your age?
  - Where do you live?
    - (prompt: City, province/state, country)
  - How do you usually describe your gender?

(prompts:

- - - Man - Cisgender (same as sex-assigned-at-birth) or Transgender (different from sex-assigned-at-birth)
    - Woman – Cisgender or Transgender
    - Non-binary – Agender, Pangender, Genderqueer, Genderfluid, Gender-nonconforming, Two-Spirit, A different choice)
  - How do you usually describe your sexual orientation?

(prompts: Gay, Lesbian, Bisexual, Pansexual, Asexual, Queer, Two-Spirit, Heterosexual or straight, a different choice)

- - How do you usually describe your race or ethnicity?
  - Do you identify as a person with a disability?
  - Do you have any other identities you’d like to share with me that I haven’t yet asked about?

What made you want to join the SOGI OTDT patient advisory team?

Could you tell me about your personal experience (or experiences) with the organ and tissue donation and transplantation system, generally?

--

*Inequities or harms related to SOGI identity*

I’d like to now ask you some questions about how your identity as a sexual orientation or gender identity diverse person (or an ally) has impacted your experience.

How inclusive of your sexual orientation or gender identity was the OTDT system?

- How competent were OTDT staff in engaging with sexual and gender minorities in your experience? How could this be improved?
- Did staff clarify your gender identity vs. your sex-assigned-at-birth at any point?
- Did you have to repetitively disclose your gender identity during the course of your OTDT system engagement?
- A lack of training in sexual orientation or gender identity and cultural humility for healthcare workers has been recognized as a gap in care for many parts of the health system. Did you have any experiences in the OTDT system that would support a need for enhanced healthcare worker training in sexual orientation or gender identity issues?
  - - - What sexual orientation or gender identity related skills do you think it is most important for healthcare workers to learn?
- In your opinion, which type of healthcare worker (doctors, nurses, transplant corrdinators, front desk staff, pharmacist etc) has the best and worse SOGI training?

What challenges did you face in the OTDT system? How were these related to your sexual orientation or gender identity?

- How did your sexual orientation (or that of your loved one) impact your experience?
- How did your gender identity (or that of your loved one) impact your experience?
- Reflecting back on your experience, did any of your other social identities (not sexual orientation or gender identity) but things like where you live, your race/ethnicity, age, religion, abilities, etc. impact your experience as a SOGI-diverse person in the OTDT system in a meaningful way?

How have the challenges you faced during your OTDT experience impacted your life?

- What ongoing challenges related to your sexual orientation or gender identity and the OTDT system are you experiencing (if any)?
- How comfortable do you feel telling the members of the OTDT team about you or your love one’s sexual orientation or gender identity information? In your opinion, what is the best way to collect such data (self-reported forms, face-to-face discussion with trained personnel)
- How worried were/are you about being treated differently if you reveal you or your loved one’s sexual orientation or gender identity?
- Do you feel the transplant institutions provide a friendly environment for sexual and gender minorities? If yes, why (examples might include welcome signs, gender-neutral bathrooms?) If no, why not ?

Did OTDT system healthcare members acknowledge any SOGI-specific inequities/harms you were facing?

- What inequities were discussed with you? Who? How?
- Reflecting back, do you think information exchange during your OTDT involvement was open and transparent?

Were you able to identify SOGI-specific inequities/harms as they were happening? If so, how did you respond? Did you tell a healthcare team member about them? What did those conversations look like?

--

***Interviewee-Specific Prompts*** *(Examples of inequities/harms that have been identified in the literature) specific to the OTDT experiences of the interviewee. Each category of prompts should only be administered for interviewees who have a lived experience of OTDT that matches (e.g., living organ donors get asked the living organ donor prompts, etc.).*

- Living donor
  - The risks of inadvertent outing during the living donation process (where the potential recipient is told that the organ they are being offered is from a donor at increased risk of transmitting HIV or viral hepatitis) have been described. Reflecting on your experience did your team address this potential risk with you at any point? If so, when?
  - For non-cisgender interviewees: did your doctors discuss with you whether your gender identity vs. sex-assigned-at-birth vs. any hormone therapies would impact interpretation of your kidney function tests – or your candidacy to donate a kidney?
- Caregiver of deceased donor
  - In your dealings with the OTDT program were you told that anything would be different based on your loved one’s sexual orientation? If so what were you told?
  - Did you provide consent for the team to examine your loved one’s body as a part of the workup for donation? Did they mention a physical examination to look for evidence of sexually transmitted infections? Did they mention an anal examination (if so the purpose of conducting this)?
  - Was your loved one declined for organ or tissue donation on the basis of their sexuality? How did this impact you?
  - If your loved one’s organs or tissues were rejected – did the OTDT program offer you any explanation why at the time of the rejection? Did they offer you any ongoing support?
  - What impact does the rejection of your loved one’s organs or tissues have on you?
  - For non-cisgender interviewees: did your doctors discuss with you whether your loved one’s gender identity vs. sex-assigned-at-birth vs. any hormone therapies would impact interpretation of their kidney function tests – or their candidacy to receive a kidney transplant?
- Transplant recipients
  - For non-cisgender interviewees: did your doctors discuss with you whether your gender identity vs. sex-assigned-at-birth vs. any hormone therapies would impact interpretation of your kidney function tests – or your candidacy to receive a kidney transplant?
  - Did your OTDT team discuss your sexual orientation or sexual practices as they relate to immune-suppression status? What did they say?
  - Were you counselled about receiving (or potentially receiving) an organ that was at increased risk of transmitting HIV or viral hepatitis? What do you remember about this conversation?
  - Did you face any barriers in getting listed for a transplant? If so did your SOGI-diverse identity impact that process?

*Opinions/beliefs on specific policies, recommendations or gaps in care*

I’d like to discuss some of the specific policies and gaps in care that have been identified as relevant to sexual orientation and gender identity diverse people and to get your thoughts/opinions on these.

- How do you think treatment of 2S/GBTQ people within OTDT systems could be improved?
- Both Canadian and U.S. health systems consider ALL sexually active 2S/GBTQ+ men who have sex with men as being at increased risk of transmitting HIV or viral hepatitis compared to cisgender, heterosexual people.
  - What are your perspectives on this policy? How could it be improved?
  - Some criticize this policy for discriminating against queer men based on their sexual orientation since not all sexually active 2S/GBTQ+ men participate in behaviors that would increase their chance of contracting HIV or viral hepatitis. Would you favor a gender-neutral, behavior-based policy instead? (E.g., one that asks people about specific high risk behaviors regardless of their gender or sexual orientation)?
  - Why do you think this policy has not been updated yet?
- Right now we test potential organ and tissue donors for HIV or hepatitis before they donate to ensure safety. There are different types of tests available, and some are better than others. In Canada we only use the NAT (the most sensitive test available) in donors who are considered to be “at increased infectious risk” (like sexually active male 2S/GBTQ+ donors). If we used this best NAT test in everyone it might mean we don’t have to even ask about sexual behaviors because all people are being tested.
  - - What are your perspectives on this issue?
- Some argue that classifying donors or organs as being at “increased infectious risk” is stigmatizing.
  - What do you think about this?
- There are different periods of time in which if a 2S/GBTQ+ man has had sex with another man they are considered at increased risk of transmitting HIV or viral hepatitis, even with a negative HIV/hepatitis test. For organ donation this is within 30 days (in the U.S.) or within 12 months (in Canada. For tissue donation this is within 12 months (in Canada) or within 5 years (in the U.S.).
  - What are your perspectives on these different policies?
  - Would it make more sense to have 1 time period?
- In our literature review we identified that there is insufficient provision of gender-affirming care in the OTDT system.
  - Do you agree/disagree? Why?
- Some say that the current informed consent processes do not cover sexual orientation or gender identity-relevant issues in enough depth.
  - What was your experience like?
  - Should OTDT systems disclose differential treatment or processes that relate to sexual orientation or gender identity? How could this be better communicated?
- In Canada the CSA recommends rectal exams in deceased male donors to evaluate for receptive anal sex. There is no standardized informed consent process to inform SDMs about this practice.
  - What are your thoughts about this?
- The caregivers and loved one’s of deceased donors have described compounding grief and suffering of finding out their 2S/GBTQ+ loved one’s offer to donate was declined on the basis of their sexuality.
  - Would earlier knowledge of eligibility criteria (not so close to the time of death) help to lessen this pain?

--

*Benefits of SOGI identity*

If its ok with you, I’d like to shift our focus now to talk about some of the strengths or good things that come from having a sexual orientation or gender identity diverse experience.

First, could you tell me about your involvement in the 2SLGBTQIA+ community?

Where did you find support during your OTDT journey? Did you find support from other SOGI folks?

Do you think your involvement with the 2SLGBTQIA+ community had any positive benefits on your journey through the OTDT system (e.g., getting an organ, supporting a loved one, donating an organ)?

Are you aware of any positives that a SOGI-diverse identity has had on people you know of in OTDT?

- Strength and resilience?
- Network benefits? (A social system of support)
- Health literacy?
- Histories of health advocacy?

If you are involved or connected to the 2SLGBTQIA+ community how did this community respond to hearing about your involvement with the OTDT system (whether you needed an organ, were caring for an organ donor, or in donating yourself)?

Do you have trust in your OTDT system?

A lack of transplant-related research in sexual and gender minorities has been recognized. Would you be interested in participating in future research to help the OTDT system better serve SGM populations? If not why?

--

*Additional considerations*

Are there any other parts of your experience that we’re missing? That you’d like to tell us?

--

*Closing*

Thank you for sharing your experiences with us today. To remind you, we will prepare a written transcript of this interview and provide it to you for your review before moving forward. You’ll have the opportunity to edit or make revisions if you feel any of your responses don’t reflect your beliefs.
