# Supplementary material for: Lived Experiences of Sexual and Gender Minorities in Solid Organ Transplantation: A Best-Fit Framework Synthesis and Inductive Thematic Analysis
Source: Can J Kidney Health Dis. 2025 May 29;12:20543581251331703. doi: 10.1177/20543581251331703 (PMC12126676; doi:10.1177/20543581251331703)
Supplement: sj-docx-2-cjk-10.1177_20543581251331703 – Supplemental material for Lived Experiences of Sexual and Gender Minorities in Solid Organ Transplantation: A Best-Fit Framework Synthesis and Inductive Thematic Analysis [file sj-docx-2-cjk-10.1177_20543581251331703.docx]

Appendix 2 Tables

Appendix 2, Table 1 – Stigma, Discriminatory Criteria & Inertia to Change

| Sub-Theme | Representative Quotations |
| --- | --- |
| 3.3.1 – Shared Decision Making & Risk Tolerance | 3.3.1.1 - “The problem comes in, is the education that patients are receiving when presented with kidneys that are at high risk. Simply saying a kidney is at high risk and expecting the patient to remember all the stuff that they got from their transplant evaluation is heading in the wrong direction already… if I'm accepting a high risk kidney, I need to know a little bit more than the fact that it's a high risk kidney. I need to know, you know, hepatitis, what do we know, there are ways of correcting that now. I have lost count of how many people I know who are living with HIV and have like zero counts. These things need to be factored into the transplantation process.”  3.3.1.2 - “I felt really comfortable getting an organ from anyone in the queer community. I have some understanding of the risk of HIV and AIDS and hepatitis and what we can do post-transplant if those are potential issues. For me it was like I'd rather live and have some risk, right? Because right now my trajectory is death. So I'd rather live. And, those just don't seem like that substantial risks in terms of all of this.” |
| 3.3.2 – Impact of ‘Increased Infectious Risk Donor’ Stigma | 3.3.2.1 - “So there was a conversation about high risk donors and that was during the transplant process… I remember at the time I was like wait like I'm also gay you know, and so that was kind of like a jarring thing to me. It was like, oh my gosh, like we're considered high risk? Also, I was a child too, you know? I was not out to my pediatric team. Whenever you're exposed to something like that as a child a kind of shapes the way of which you view your identity, so that was not really a great thing to experience.” |

Appendix 2, Table 2 – System & Structural Elements: Testing & Inclusive Care

| Sub-Theme | Representative Quotations |
| --- | --- |
| 3.4.1 – Enhanced OTDT Healthcare Provider Training | 3.4.1.1 - “Better training you know in medical school and nephrology, you don't get a lot of training on working with LGBTQ+ patients in general. And I've helped with some of the trainings. I've been a patient where people can ask questions. And I'm always amazed when I ask them how much training or interaction they've had with LGBTQ people. They’ll say things like, ‘oh, well we've read a chapter about LGBTQ people’. Or you know ‘we had someone come in and speak one day’ and it's so minimal. A lot more of that training is needed. We have some antiquated policies around blood donation and organ donation that don't make sense in terms of men having sex with men or sometimes trans men having sex with men are included in that, sometimes we're not, you never know. Trans women are often included in that because you know, of the HIV and AIDS crisis, and the stigma around that being an LGBTQ+ disease.”  3.4.1.2 - “Being able to have frank discussions with patients about different concerns that they may have that are specific to their sexual orientation or to their gender identity is important. Being able to create an environment where people are able to share about their identities is important, so that when there are issues that come up that they feel comfortable talking about them, that they know that they're an affirming and supportive environment. At the same time acknowledging our limitations and not trying to overstep and say that we have all of the answers. I think that there's a potential for harm if we say that we are an affirming environment, that we're welcoming, but then we make mistakes. When we don't acknowledge where those mistakes are... I guess that would fall under like a sense of like cultural humility and the ability to be corrected and when we make mistakes and to learn from those mistakes.”  3.4.1.3 - “Having signs and everything is great, but there also has to be the training to go along with it to make sure that the staff members and providers are appropriately trained and comfortable in providing inclusive care. Because the worst thing would be that you present this outward facade that you're welcoming, but then when the rubber meets the road, the actual interactions are not actually affirming. That could potentially be very damaging. These efforts need to occur in parallel. But I think both are important.”  3.4.1.4 - “Do transplant providers provide a friendly environment? I'm gonna say overall no. But I've had really good experiences. The places I've been to have been really deliberate around doing pretty extensive training. They're all still hubs for LGBTQ culture. So, my experience is not at all universal. Most people do not feel recognized or understood or validated in their identities in these spaces.”  3.4.1.5 - “I recall a lot of this, sometimes referring to my husband as my ‘partner’. I just kind of remember going through this process and I would correct them and say ‘husband’. He was my partner as well, but he was first and foremost my husband. I did feel as though it If this was a different scenario, someone's wife wouldn't have been called a partner. I just knew that some people were struggling with how to identify our relationship... so it was just kind of that whole husband-partner dynamic that I struggled with a little.”  3.4.1.6 - “something that I always like is when people, if I bring up that I'm married, that they don't automatically assume that it's a ‘she’. Because the amount of relief that I feel when someone refers to my partner as a ‘they’. They're leaving it open, right, and they're not just assuming. That to me is like, ‘okay, I can feel comfortable around you now because it's not just a heteronormative box right’.”  3.4.1.7 - “Learning how to use peoples’ correct pronouns is important and referring to them in the way that they want to be referred. Humility of understanding people's identity as being very personal and however somebody identifies we need to be able to respect that. And not only acknowledge it but also celebrate it as well.”  3.4.1.8 - “We often talk about the ‘trans broken arm syndrome’. So don't assume everything we have is because we're taking testosterone or taking estrogen or because we are trans people. We can break our leg and it's not really testosterone or estrogen, or us being trans people. You know with kidney failure taking testosterone does increase creatinine a little, but it doesn't impact kidney function in the way that some people think it would impact kidney function, so it's a safe thing to be taking even if you are in kidney failure.” |
| 3.4.2 – Institutional Action | 3.4.2.1 - “I felt like my nephrologist was really good about it. He would be like ‘this is what the policy says I don't necessarily agree with what the policy says, but I have to tell you what the policy is and what we have to abide by, but here's what I think’. And so I think like you know, being able to communicate this and we are working on addressing equities in terms of policy. Instead of just being like, ‘okay, here's the policy and because of who you are you can't donate or you're at any increased risk’.”  3.4.2.2 - “I think that there are many barriers that exist and so there needs to be extra outreach needed to overcome and to compensate for those barriers that are there.”  3.4.2.3 - “Where I get my care now, they've only just within the past couple of years begun participating in pride, marching in the pride parade, having more messaging and more events during Pride Month. I think that can be important to show that it that the institution is welcoming for people with different sexual orientations and gender identities. “  3.4.2.4 - “For me it’s really affirming to watch this transformation and even what we've seen come out in the literature in the past 6 years from when I had my transplant is exciting, and there’s new things all the time. And being part of that and seeing that is really affirming.”  3.4.2.5 - “From a policy standpoint, I think it just needs to be a non-discriminatory policy first, about not just your sex assigned at birth, or your race or color, or your religion. Your gender identification will play a role in this, how you identify is also, you know, not grounds for discrimination. That simple addition changes the game because it now makes it something that the doctor has to think about, and that the nurses have to think about... there are implications if I don't follow this policy.”  3.4.2.6 - “Standardized procedures like rules, guidelines, and making sure that all healthcare workers know what those are. That's…and to me that's just simple, that's what should be happening. You would think this would be standard procedure.”  3.4.2.7 - “She had gotten a kidney in (the same province where my son was declined as an organ donor) about a year before, and she said ‘I have to tell you it was from a gay man and it was disclosed to me beforehand whether I wanted it or not’. And from what I understand, that's what proper procedure should have been in (my son’s) case. And that's when you know, when all these healthcare workers and people started coming forward, that's when I really realized there was…there was just a total lack of standard procedure in every province that communicated with me across Canada.”  3.4.2.8 - “They definitely acknowledged that there's not a lot of data on LGBTQ identities and trans people specifically. I've been set up almost instantly with a climate person, so if you have any problems at this institution relative to your identity there's a number that you call and this person can help you… and they will be an advocate. That is really affirming because sometimes as a patient you feel like especially when you're hospitalized like you just kind of have to go along with whatever.”  3.4.2.9 - “(We) started a LGBTQ+ transplant Facebook group because we've noticed the increase in discrimination in other transplant groups. (We would) post articles about transplants and LGBTQ+ people and that would infuriate people. So we're like, let's just start our own.” |
| 3.4.3 –  Trust in the OTDT Health System | 3.4.3.1 - “When I was going through the transplant process I was… I will be honest, I was very worried about compliance. So I was trying to do all the things I needed to do. I was reaching out to people outside of that group to ask questions about ‘what do we know about hormone use and kidney function? What do we know about trans people and transplantation? What do we know about these things?’ And so I didn't ask my team a lot of things until after the transplants happened because I was worried about compliance stuff. Did we have some of those conversations? Yes, but I was very, I was careful in that process until after the transplant happened for sure.”  3.4.3.2 - “There was this really big fear that If I came out that (my transplant team) would start viewing me differently. Maybe not everyone might think this way, but I am essentially the child of immigrants. I am a heart transplant recipient and I am a person of color too and so I feel like there was…in my household, there is medical mistrust and that might seem a little weird because my life was saved by doctors, but I always feel that there's this gap between me and doctors where there's this hierarchy and that can't really be bridged. I know that doctors don't think this way. They've tried to say things like, ‘I communicate with the patient, like the patient knows that they can ask me anything’, but really there is this gap between me and the doctor and I always felt that kind of uncomfortableness there. And so adding on to this thing like sexual orientation, another minority status, felt really like something I didn't really want to do. It might be different for others, but for me, I felt already discriminating against, so I didn't really wanna be on top of that.”  3.4.3.3 - “As a transplant patient, you can feel that pressure even more because you are being evaluated based on being compliant. So if you are not compliant in something because you don't want it to be done to your body… you constantly feel afraid.” |

Appendix 2, Table 3 – OTDT Patient & Community Relations

| Sub-Theme | Representative Quotations |
| --- | --- |
| 3.5.1 – Informed Consent | 3.5.1.1 - “If you have informed consent forms, people need to be walked through what they're signing, and then they need to also make sure they have appropriate language in there. And I would wager that most of them do not include this in their informed consent forms. And even if they did, they probably didn't not walk the patient through step by step, exactly what they were consenting to. And I don't think I've ever… I mean even you know you're in the ER the hospital wherever, they give you these forms, but they don't give you the time to read them. Or to understand them, you know. It's like we're taught anything you sign, a lawyer or someone should look over first. But yeah, we're in these situations, and the expectation is just sign it here. Just sign it.”  3.5.1.2 - “The problem comes in, is the education that patients are receiving when presented with, kidneys are at high risk. Simply saying, a kidney is at high risk and expecting the patient to remember all the stuff that they got from their transplant evaluation is heading in the wrong direction already. Because it has to be… if I'm accepting a high risk kidney, I need to know a little bit more than the fact that it's a high risk kidney, I need to know, you know, hepatitis, what do we know? There's ways of treating that now. So… HIV. I have lost count of how many people I know who are living with HIV and have like 0 counts. These things need to be factored into the transplantation process.”  3.5.1.3 - “They did not discuss that with me actually and in retrospect that didn't come up. Being that I also attended most of my husband's appointments as well, I do distinctly recall the conversation with the doctor who remarked to my husband, ‘to clarify, are you aware that your donor has a pineapple allergy?’ Which he looked very exasperated and having to do, knowing that we were married and my husband is very much aware of my allergy, which is anaphylaxis unfortunately. So I could see the exasperation in the doctors expression having to deal with that. But in terms of increased risk for transmitting HIV or viral hepatitis that was not a question that came to me and it was not covered on my husband's end.”  3.5.1.4 - “I could have been more concise on my end, being that I never openly discussed my bisexuality. Not for the attempt to be purposefully avoidant of the topic, but because my monogamy established that my orientation and behaviors carried what I would have deemed a minimal risk, and wouldn't have played a factor in long-term care, especially after donating. I feel maybe I could have been more open, but if that door had already been opened, I feel that I would have walked through it. So if somebody had mentioned my, you know…do you want to identify as SOGI diverse? I have no reason to hide that. I've had no reason to hide in the past. So I feel if that door was opened from their end, I would have stepped through.” |

Appendix 2, Table 4 - Benefits, Strengths & Resilience of the SGM Community

| Sub-Themes | Representative Quotations |
| --- | --- |
| 3.6.1 –Accessing Transplantation | 3.6.1.1 - “When I was diagnosed, I was really obviously upset with being diagnosed with end-stage kidney disease. I was really upset about what my timeline would look like, what my life would look like and my family was initially concerned about telling everyone and how people would think what people would think about me or any number of things. My queer family was completely the opposite where they were like ‘well we need to broadcast this, we need to find a donor, we need to figure out how we're gonna pay for this’. Like it became like this is a community thing that we're gonna do and you know a lot of people had said things like that because I did so much for the community and helped so many people that they wanted to see me succeed and that this was going to be a community effort. So an old friend set up a Go Fund Me account and started raising money so that when I could have a transplant we would have the money ready for that. And then you know, I put it out there. I said, ‘Hey everyone look, I have kidney failure. I'm going to need a transplant. Here's a link to the site where you can get screened for potentially donating a kidney’. So I literally just put it out on Facebook. And I had over 70 people sign up who were willing to donate.”  3.6.1.2 - “Anything that expanded my social network I think was in a way beneficial when I was looking for a living donor. I ultimately ended up receiving a deceased donor transplant, but when I was trying to reach out to my networks, there were many people that came forward. Some of whom were through professional and other personal networks, some of whom were through networks of people that I knew through engagement with different queer groups and organizations.”  3.6.1.3 - “Yeah, I think that there can be an increased sense of community and shared experience within the queer community. There was somebody in the gay men's chorus here that donated a liver to another member… I think it's just in the queer community, recognizing the importance of community, and found family beyond just our shared experience. I think those are all important things that can drive people to become organ donors to people that they may not know or may not know well.”  3.6.1.4 - “I hope that I keep this kidney forever but if the day comes where it's no longer viable, I hope that my community here would help me.”  3.6.1.5 - “In my situation what I will say is that my family was more on the page of like well, let's not announce this, we don't want to tell everyone all your business. My queer family was like, oh no, we need to announce this everyone needs to know, we need to figure this out. My partner and I, we do a lot of research around, queer political culture and talk about how because of the AIDS crisis we have a lot of embedded… How do we engage in politics and social movements and different things. And we transfer that into our community as queer people. And so we saw that like everyone was like, no, this isn't gonna happen, we're gonna help you.”  3.6.1.6 - “Being Jewish also has its own privileges. There's a great organization run by an Orthodox organization. They do a lot of networking stuff to get people kidneys. I was going through them, but then I got a cadaver donor. They do a lot of connecting and you know, there's privilege in being able to do that.” |
| 3.6.2 –Sex-Positive Culture as a Facilitator of Sexual & Reproductive Health Promotion | 3.6.2.1 - “I felt like within the queer community we are open about who we are, who we’re with and what we're doing. Whereas I have a lot of straight friends who are doing a lot of the same things who aren't… their culture and community doesn't allow for that openness. And so I can see the risky behaviors happening and hear about the risky behaviors, but there is not the openness that there is in the queer community to be able to say, yeah, these are the things I engage in or don't engage in.” |

Appendix 2, Table 5 – Transgender & Gender Diverse Considerations

| Sub-Theme | Representative Quotations |
| --- | --- |
| 3.7.1 – Gender Identity & Health Information Systems | 3.7.1.1 - ‘No, no, no! Out of the 2 transplants that I've had, it's never been a discussion.’  3.7.1.2 - “…that would have been again, the provincial health care number system. Absolutely, M or F. Just one or the other.”  3.7.1.3 - “I think improvements could look like… being a space that feels a little bit more welcoming and open to people of all different types of sexual identity and gender identity. I also believe that asking people about their sexual identity and gender identity in a questionnaire or in person would be a great step.”  3.7.1.4 - “Every time I'm at (my transplant center) I feel like I'm asked about pronouns and it's usually a confirmation, ‘okay, you were assigned female at birth. How do you identify now?’ They don't even ask with an assumption. They literally ask how do you identify now and then what are your pronouns and it's pretty well every time.” |
| 3.7.2 – Material Considerations | 3.7.2.1 - “I also had a kidney biopsy done at the local hospital, and that was a really horrible experience… So they put me on a floor with other men, fine. I had to share a room with another guy. Sharing the room was not comfy in general and as a trans person is really not comfy. And then, the problem that occurred is that all of the equipment that they brought me to urinate was like one of those things that… I was like ‘I can't use this’ and they were like, ‘well… you just, you have to use it’. And I was like, ‘well I can't, this isn't gonna work’. And they were like, ‘well, this is what we have for you’. And I was like…’what?’. But the weirdest part is I was on this floor with men, they were treating me like other men, even though I didn't necessarily have exactly the equipment and then they were still messing up my pronouns.”  3.7.2.2 - “I don't think there are gender-neutral bathrooms. I think in the way the floor was divided, of girls rooms and boys rooms, I think that was definitely a pertinent thing. And I also think that in terms of the activities in the hospital, it was also a boy versus girl type of thing. So I would understand that for someone that's gender queer or someone that is non-binary that might be an issue for them.” |
| 3.7.3 – Opportunities to Enhance Care for Transgender Patients | 3.7.3.1 - “I'm in contact with some parents of children who are outside the US trying to figure out how they can both support their child's gender identity and deal with chronic kidney diseases and feeling like neither group is really having a conversation around how to best support them. It's been a privilege for me to be out about my trans status and my transplant status and that there are a lot of people out there who can't be out for various reasons, and who are struggling to be able to find affirming care.”  3.7.3.2 - “I feel like it's often presented as like ‘we don't have the expertise, we wanna make sure you get the right care’, when behind the scenes it’s really ‘I don't necessarily feel comfortable with you’. And we as trans people, we know that… we see it. We see you when you say that, right? Because what you would do if you felt like you didn't have the expertise or you didn't have the knowledge base? You would actually find someone and refer us to that person and help us on that journey instead of just putting your hands up in the air and saying, ‘look I can't help you’. The majority of us as trans people, we know what you're doing when you say ‘hey, I can't help because I'm not an expert’ and you don't.”  3.7.3.3 - “I have friends who are other places that have found it really challenging, really impossible, to find a nephrologist who will work with them because they don't have an expertise in trans people. I do have a friend who's having a hard time even finding a nephrologist who wants to take his case.”  3.7.3.4 - “When I started the process every time I would go to the doctor I'd have to do this long explanation about being trans and what that was like and I just… I almost was like, I just wanna die. Just let me die because this is too much to have to like have to explain my identity and go through this.”  3.7.3.5 - “I remember having a conversation with the Transplant Coordinator. And I was like look, I don't know that this (renal function) calculation is correct and we need to have more conversation about this calculation. And she said to me, “it sounds like you're really trying to get an organ transplant and when you get an organ transplant, this is a lifelong thing and you're gonna have to take care of it and you’re not gonna want one sooner than you need one”. And it was so dismissive and she and her last comment was “and we don't even know if you'll be able to get one”. I felt like I was often trying to ask questions that they didn't think a patient should be asking or telling them things that they didn't think a patient should be telling them.”  3.7.3.6 - “I have been out and what I think has been hard to see is the number of trans people around the world who have contacted me about their own issues with chronic kidney disease and feeling that they haven't been heard. They haven't been seen, that they've been taken off testosterone, taken off estrogen asked to de-transition. They’ve been told that they couldn't have a transplant unless they de-transition, told that they could never transition if they do get a transplant. So the number of people who are experiencing those issues and have limited access or no access to quality affirming health care is really upsetting.”  3.7.3.7 - “The first doctor I saw was a local doctor and she had never worked with anyone who was trans. She wasn't sure if she'd ever work with anyone who was queer in general. And it was a really challenging experience because she immediately told me that I need to go off testosterone. And…so I went off the testosterone, which was catastrophic for me. In dealing with going through and coming to terms with organ failure, and then not having hormones… it was really, really problematic.”  3.7.3.8 - “There was no conversation around the risk and benefits in terms of stopping. The Nephrologist communicated back to my doctor at the health center that this was her recommendation. So my doctor wouldn't prescribe any more hormones. So I was I was cut off.”  3.7.3.9 - “When I was in a rural community and accessing rural healthcare, it was horrible. It was so bad that I felt like in some ways I’d rather die than go through this process of being, feeling humiliated and not exactly ridiculed but having that…overhead. My wife and I decided that we would have to live reasonably close to a major metropolitan area and only seek health care from a major university centre. We also decided that we can't live in a conservative or red state. When we were on the job market, we had opportunities in Texas, we had opportunities in Florida and that was no-go. That was an absolute no-go because of my health and my trans status, those are not safe destinations for us.”  3.7.3.10 - “I'm extremely worried in general because of the experiences when I'm not at a university healthcare center. I haven't had the best experiences at our local healthcare centre so now anytime anything happens, we will drive for an hour and a half, even if it's just to go to the emergency room. And I have a document that says if I end up at our local healthcare centre to airlift me out. Just get me out, don't even try, just get me out. Because I don't feel like I can get adequate care because of my trans identity.”  3.7.3.11 - “I do love to travel and I will tell you that there are many places in the US right now that I will not travel because I don't, I don't trust the ERs, I don’t trust the hospitals.”  3.7.3.12 - “You expect to be treated with respect as a patient in general. I have a classmate from high school and her husband is a doctor in Florida and he's very conservative and he doesn't believe trans people should exist. He supports the idea that he shouldn't have to treat LGBTQ people. And he's at a major institution in Florida. So that personal connection tells me that it isn't safe and there's a lot of other people out there who feel the same way. And so at least in Florida right now we're in the process of giving license to people to be able to discriminate. And that's terrifying. Absolutely terrifying.”  3.7.3.13 - “I had one nurse literally tell me she doesn't agree with transgenders, like that's how she framed it: “I don't agree with transgenders, but I, you know, I'm here to help you”.  3.7.3.14 - “(My transplant physician) acknowledged that there's not a lot of data on LGBTQ identities and trans people specifically, so there's definitely that acknowledgement.”  3.7.3.15 - “I did face barriers being listed. I did face the barrier in how we were calculating my kidney function. Where I was you had to be at 20 or below. And based on the male numbers, I was at one point, I think it was like at 22, but that we weren't sure that that was accurate. And so there was a lot of back and forth about when can I be listed? When does it make sense to transplant?”  3.7.3.16 - “It was a repeated challenge. I think the hard part is that most places have a designated kind of cut-off for being listed and what I understood at the time is it was hard to overwrite the system. Your cut-off is based on how your bloodwork is entered. By the time I had port placement for dialysis, I was down to I think it was in 8 for kidney function on a male scale. And I was down to I think 91 pounds at the time. It was pretty rough and when I had the port placement I had a significant bleed, almost died in that process. They had to do emergency dialysis, I was in the hospital for several days. That was a pretty tense process. It was unclear because there was no literature about what exactly to do and there still isn't a lot of literature. We're still trying to develop that, right? It was unclear based on my body type, the sex organs I do have, the hormone levels… all the things we needed to figure out and factor into this equation. We waited as long as possible to do dialysis and I think we both recognize that we waited too long.”  3.7.3.17 - “Now we know because of muscle mass and all these other factors, we know that that's really problematic, especially because in all my documentation I'm coded as male… on my birth certificate, driver's license, everything is coded male. So everything is run as male, but if you take away my hormones, then that doesn't that doesn't work anymore. So it's really a problematic piece where then, I could do these calculations and know I'm not gonna be put on the transplant list when I need to be put on the transplant list because you're calculating my numbers wrong. And it was a very scary time in terms of figuring out like how do I… how do I communicate this to my doctors? And I'm not a doctor, right? Like how do I tell them this and get them to listen because they're not really listening?”  3.7.3.18 - “I shouldn't have to be reading medical journals to understand my own health because I'm not getting proper care.”  3.7.3.19 - “For transgender patients… I mean it's sounds absolutely crazy that I would be calculating these numbers, but for trans patients we often have to know…we often have to do a lot of research into our own care in order to get adequate care. And it can be really frustrating and life-threatening in some cases.”  3.7.3.20 - “In the ideal world, I would love to see that across institutions we could receive equal care. And that's not the case. I think that's a really sad statement. So I'd like to see that happen. I'd like to see that we continue to work on equations to better identify how we assess kidney function in the trans and non-binary community. But then that also of course translates into communities of color and other communities as well in terms of these equations like, this one size fits all system doesn't necessarily work.” |
| 3.7.4 - Benefits of Gender-Affirming Care | 3.7.4.1 - “In both (my transplant centers) I've been set up almost instantly with a climate person so if you have any problems at this institution relative to your identity, here's a number that you call and this person can help you and please be assured we've all been trained. So they tell me how they've been trained and who's been trained and what, you know, all the things and then if you have any problems, call this number and they will be an advocate. And that is really affirming because sometimes as a patient you feel like especially when you're hospitalized like you just kind of have to go along with whatever.” |
|  | 3.7.4.2 - “At my current transplant institute they immediately put me back on hormones. I remember the first time talking to a transplant doctor. I was like, ‘hey, so look here's the deal, I'm a transgender guy I've had hormones for almost 20 years. Here's what's happening’. And she was like ‘okay’. And I was like, ‘wait, do like, did you hear me? Like I am a trans guy. And so I may need different care.’ She was like ‘okay’, like it was like nothing. And that was repeatedly my experience at my current hospital. Which was wonderful. It's absolutely wonderful.” |

Appendix 2, Table 6 – SGM Priorities, Harms & Opportunities for Improvement

| Sub-Theme | Representative Quotation |
| --- | --- |
| 3.8.1 – Sex-Positive Sexual & Reproductive Healthcare | 3.8.1.1 - “I think that's important, especially if you're going into the realm of STDs and getting screened for HIV and your panel, I think that is relevant. You need to keep yourself safe and so being able to talk to your healthcare provider about your identity and your sexual orientation and who you're having sex with is important.”  3.8.1.2 - “I am Gen X and so I've been dealing with kidney disease now for almost 20 some years, and since my first introduction to kidney disease sexuality was not discussed at all. When I went in for my transplant evaluation, sexuality was not talked about.”  3.8.1.3 - “I found that none of my physicians asked about my sexuality. None of them, none of them ever engaged in my sexuality, and these are my transplant teams. These are my nephrology nurses and doctors and the transplant evaluations team. None of them.”  3.8.1.4 - “There just isn't much… that's dealing with LGBTQ issues as it relates to kidney health. But understanding that people who sit in the dialysis chair, people who get transplants also have a sexual identity. In fact, sexual identity is usually not even discussed for the most part.”  3.8.1.5 - “Now that I’m thinking about it, I don't think my team really did talk to me about my sexual health. And being immunocompromised, I don't think they asked about what kind of sex I was having, what kind of protection I was using.”  3.8.1.6 - “There was none. Not just about sexual orientation or gender identification… There's no talk from any team that I've ever had about anything, slightly sexually oriented. It has just been completely omitted from the conversation.”  3.8.1.7 - “They'll talk about impacts of Prednisone on weight. And you know how you're gonna have to take it and why you needed it. There they talk about cellcept and technical impacts on the body. There is no conversation about how as an immunosuppressed person that puts you at higher risk for transmission of sexually transmitted diseases. You have to be more careful about who you select as your partner. None”  3.8.1.8 - “No, that's never been explicitly brought up. I've had a little bit of discussion about that with my primary care doctor, but again, no real directed discussion about sexual health or sexual practices, risk of different activities, anything like that.”  3.8.1.9 - “There was not like a whole presentation or giving me education or anything… sexual education for transplant patients who are gay? There's nothing like that. Nope, never. Done my own education.”  3.8.1.10 - “I also felt that any medical issue I had, they really didn't know what it was. So, for example, I asked to be on PrEP, and they had no idea what PrEP was and they were so hesitant to put me on it. They had me go to my hematologist and ask them if it was okay. It was viewed as this non-necessary type of medication that I shouldn't be on... I was just like, ‘I really don't want to get HIV’. Like, for being immunocompromised, I really don't want to get HIV. To this day I'm still not on PrEP. That wasn’t a really great experience to go through where I had to basically beg my doctors to put me on it.”  3.8.1.11 - “Trust is kind of flip-floppy to me. I trust (my transplant team) in terms of decisions about my health, what they say about my health, transplant facts. I can fall back on my transplant team if I have any medical things that I need to take care of. I know that they're on it and I trust that. In terms of trust and my personal things, and I consider my queer identity a part of my personal life, not really. Never really has been in all honesty. I think that my care team provide some distance between me and them… If I do have any questions about how to have sex again or if I would like to have sex again. I would obviously tell them, but I don't think I would get a very good reaction from them. I don't think that they would be supportive of it. And I think that they probably wouldn't want to talk about it either because they're like, ‘sex, that's a personal thing’, like not for you, but then It kind of is like a medical thing as well because the HIV prevention is so, so important someone that’s immunocompromised. And so overall It's complicated.”  3.8.1.12 - “What I discovered is that it wasn't so much my sexuality that they didn't want to talk about. They didn't know how to talk about sex, period. I've never seen my nephrologist blush as much as when I asked him about sexual function and kidney disease, and how I would like to be sexually active. And I swear he turned multiple shades of red right in front of me. It was like, ‘okay, I'm asking you this question but it's obvious it's not my sexuality that you’re nervous about… it’s that you don't know how to talk about sex. So that was an interesting experience.”  3.8.1.13 - “Sometimes people ask if you're sexually active. Like, are you sexually active? Yes, I am. Do you use protection or a contraceptive? I'm like, no, I don't. They're like, okay, you have unprotected sex with how many partners? Any risk of being pregnant? I'm like, no, absolutely not. They’ll ask ‘are you sure?’ So there's those times where I get really hounded about pregnancy. It is not possible. The sex that I'm having is not possible for this to happen because it’s with a woman and usually once I say that they're like oh okay we finally get it. And then other times they actually read my chart and they're like, oh, lesbian. Okay, great. So we don't have to worry about a pregnancy test. So it happens and that's really frustrating.”  3.8.1.14 - “I think that trust kind of waivers when we get into sexual and gender identity. There’s some work that needs to be done there and education with healthcare providers in general. Eventually things will change and they'll come around. People my age now are becoming doctors and nurses and then Gen Z will be doctors and nurses and we're kind of changing the guard there. But I also don't want to wait for that to happen. So implementing change with the people who are still there now would be great and that includes educating them.”  3.8.1.15 - “Internalized fear maybe even some internalized homophobia about my identity and my sexual health and my sexual identity made it difficult for me particularly at the beginning to share all these details with my transplant providers. Of course, it's very important for any doctor, particularly transplant doctors to have a full picture of their patient's identity in order to provide the best and optimal care for them. And whether there be barriers on like the provider side or on the patient side, when there's that kind of disconnect or something that's preventing someone from showing their full authentic self and sharing openly, that can be a hindrance to care. I've been fortunate that I've lived in major metropolitan cities, very liberal places. So, I knew that as I became more comfortable having these discussions with my doctors, not just my transplant doctors, but my primary care provider and just in general and being more open and out about my sexuality in all aspects of my life, I've been able to have more frank and open discussions with my transplant providers specifically, but all with all my doctors in general.”  3.8.1.16 -“I have felt that I have to drive a lot of those things and I'm very fortunate that I have a lot of knowledge in this area and I have the privilege and expertise to be able to navigate the limited amount of literature in this in the space, but that's definitely not the case for the majority of people.”  3.8.1.17 - “I think a lot of times still though, it doesn't come up explicitly unless I bring it up. And that puts a lot of the onus on the patient to kind of talk about what their concerns are about. And sometimes to educate providers about different kinds of concerns that are specific to gay men. For example things like PrEP use, different considerations that we as transplant patients might need to have around different kinds of viral STIs even. Having a transplant, we're at risk for more complications for different kinds of infections.”  3.8.1.18 - “In looking the through literature, there's not really great guidance for the screening of HPV driven malignancies in men who have sex with men and with transplants in particular. There's some guidance for initial screening in the general men who have sex with men population. There's guidance for transplant patients with uteruses to look for cervical cancer. But again, there's this intersection where there's maybe not data to drive guidance. I felt like I was in a no man's land of trying to figure out what the follow-up was gonna be and discussing this with my care providers.”  3.8.1.19 - “There was this thing with the monkey pox vaccine where it was viewed as unnecessary. (My team) didn't really think it would be very useful and I kind of really had to push it to say it is something I really do need to be aware of because I had contact with someone that had monkey pox and it was so so scary. I was like, ‘this is insane, like I need to get the vaccine ASAP’. And my care team was like, ‘why?’. And I told them I had contact and so it was just this very weird dilemma where I was immunocompromised and they would be so scared of like a tiny speck of dust but they didn't care about the monkey pox vaccine. And so I did have to push it.”  3.8.1.20 - “I don't think (trans-specific issues) were something that my team necessarily brought up or highlighted. There’s kind of a standard question I ask every time I have to have a procedure or anything like “how will you take care of me as a trans patient?”. And so whenever we're doing pre-op anything or whatever, that's kind of my standard question. Instead of someone telling me how they're gonna take care of me as a trans patient.”  3.8.1.21 - “I remember I told my care team I needed the monkey pox vaccine because I was having sex… and it was kind of like the slut-shaming type of thing where they said ‘you as an immunocompromised person was doing this??’ I wasn't having anal or oral sex, so there was no way for me to get HIV because I knew how to be safe, but there was just this intense slut-shaming coming from my care team on being a gay man. I don't know if they would have reacted this way if it was someone that was straight, but they basically put a ban on sex for me. I don't know that my care team is supposed to do that, but they did. They said if I ever had a boyfriend or if I ever dated, I just need to have one person to have sex with for the rest of my life. And it's really weird when medical staff say things like that, right?”  3.8.1.22 - “I feel like we're all allowed to make mistakes… but there's just… I guess fear within the team that anything that you do will cause your immune system to be compromised because we're so, immunocompromised. And if you add on this thing like having sex, you know…being at risk for HIV, they're really, really against it. And so they don't really have, like any education. They're very dogmatic, you know, they're very much like ‘if you can avoid it, then just avoid it’. But they don't realize like it's kind of like… having sex is kind of important. And they don't really have any alternatives and so overall abstinence… I'm following what they're telling me to do. But I just kind of wish that like there was another way.”  3.8.1.23 - “I have another friend who because of this current Nephrologist… So he has been trying to get on testosterone for probably 15 years and he has chronic kidney disease and he's been denied taking testosterone because they're worried about kidney function. So even though we know in the literature that taking testosterone doesn't have a negative impact on the kidneys…”  3.8.1.24 - “I've had several incidences where they were fine with pronouns, but then they were inappropriately asking me questions, asking if I'm planning to have more surgeries, what I think about phalloplasty… It had nothing to do with anything related to why I was there. I had a traveling nurse who had never put in a catheter and wanted to try to put in a catheter on me. I needed a catheter for a situation and I was like “No, this is not… like I'm not comfortable with this” and then like the lead doctor was like, “oh, it's not a big deal, she'll be fine with this, we'll just…” and then she put it in the wrong spot. And that was really horrific.” |
| 3.8.2 – Knowledge & Practice Gaps | 3.8.2.1 - “I just know every 6 months that I go in you see the transplant center, the test (HIV serology) is gonna be done automatically. They don't ask me. It's just part of the regular workup, and to a degree very satisfying, because I think I know my health status and my numbers better than some people who don't have anything wrong with them at all. At least I know where to stand. And so it comes with Its benefits, too.”  3.8.2.2 - “It's difficult for transplant providers, as being very subspecialty, to necessarily become the experts or be the ones to do be doing everything, that may be better suited under primary care, but I think there are specific intersections where for specific issues there could be a role. For example, like I mentioned these risks for these different virally mediated STIs that transplant patients may have more adverse outcomes from. HPV like I mentioned, HSV etc. Being well-versed in things like PrEP, because there's the potential for interactions with medications, so knowing about that and bringing that up with patients. Not just sexual orientation minorities, but everybody, to be able to have that discussion with all sexually active individuals and being able to offer that and know when that would be potentially beneficial for different people. I think it's important. I think especially for me, I never had conversations about PrEP use with my transplant doctors, but it was a huge area of concern for me because of a potential nephrotoxicity of Truvada in particular and trying to weigh the risks and benefits of PrEP use.”  3.8.2.3 - “Sexual health is an important part of sexual orientation and my biggest barriers have been in having frank discussions with my transplant providers and with my primary care provider about sexual health. Again, part of that is probably my internalized fear about discussions about sex and sexual health, maybe some also internalized homophobia… That fear of having been in the closet and having like these more frank discussions with my primary care doctor and my transplant doctor. Where this has come up has been related to STIs and viral STIs in particular. For example, there is guidance for HPV vaccination. When it first came out and was approved, I didn't qualify for it, but then they raised the age limit. It took quite a lot of like coordinating between my primary care provider and my transplant doctor to try to coordinate getting that vaccination series. And then after a year having like go back and forth advocating for this vaccination and why it was important and for me individually, eventually I was able to get it. Similar to the COVID-19 vaccine, we know that people don't always have full immunity after vaccination when they're on immunosuppression so there was that concern about whether or not I would have a response or not. And in fact, on like a pap screening after my vaccination, I did find that I had positive testing for one of the HPV strains that supposedly would have been protected by the HPV vaccine, but, I did not have protection and I had the infection. So that adds another layer of things to kind of keep up with.”  3.8.2.4 - “I've heard that even from female transplant friends that have been dealing with cervical cancer that they were never told that this was something to be concerned about. But when we know that transplant patients have a lot of issues with virally driven infections and particularly virally mediated cancers, and so I think this is probably an area that requires more attention in general.”  3.8.2.5 - “I get most of that screening through my primary care doctor. But they often times are uncomfortable with a lot of the transplant related stuff. So it's a lot of ping-ponging back, deferring back to my transplant team being like, “we're not entirely sure” and trying to navigate and coordinate between those two teams.” |
| 3.8.3 – SGM & OTDT Community | 3.8.3.1 - “As a gay man with a with a kidney transplant, I've always sought out community of others with similar lived experiences. I felt like when I was in gay spaces I rarely ran into other people with transplants that were able to identify with similar experiences that I went through, or even with any kind of chronic illness. Many times images we see in the media of what it is to be a gay man out in the world, like at some circuit party or something, are very image driven and this quote unquote like picture of health kind of thing. I think this has roots in the HIV AIDS epidemic.  But I never saw myself represented, whether it be my chronic illness or oftentimes being an Asian American, I think I am oftentimes excluded from portrayals that are seen in the media. So at the same time, on the transplant side, even back to the time when I was first diagnosed with kidney disease, when I knew that I needed to be a get a transplant, I sought out people that were going through similar experiences.  Being a young adult dealing with waiting for a kidney transplant, it was difficult for me to meet other people that had similar experiences. Most of the people that I met when I was in clinic tended to be quite a bit older and didn't have, a similar kind of stage to what I had.  Eventually I found community in different volunteer organizations and Facebook groups, online forums. But again, in those places, I didn't meet other people that identified with my queer identity there, and at times encountered outright hostility. For example, when posting information about why it was important to have gender affirming care, intersections with transplant care or kidney care or things about Pride Month, it was met with outright homophobia and homophobic comments in those Facebook forums.”  3.8.3.2 - “I did quite a bit of trying to educate people about why their views were homophobic. And why it was important that there were discussions about the experiences of LGBTQ people within the transplant and organ donation kidney spaces. Because of that, I got together with a friend of mine who's also a gay kidney recipient. We formed a separate Facebook group. I invited a bunch of people that I knew fit into (an LBGTQ) identity, and posted again that we created this space. There are not that many of us in the group, but it's just nice knowing that there is somewhere that we can go that have like-minded people where we're not gonna be met with outright homophobia or transphobia.”  3.8.3.3- “I had a transplant several years ago, I think it's been about 6 years at this point. A kidney transplant, and when I was going through end stage renal failure I looked for other people like me in the literature, in the community, and it was very hard to find people. And so for me, being involved in this community where we can raise awareness that transplants are needed within our community, that we have to raise awareness about what this looks like is a really important issue for me.”  3.8.3.4 - “I remember there was a period of time that I wondered there were any other gay people who had kidney disease during my first transplant, because it's not something that people talk about. It's not an issue that you really are addressing or talking about. You're just trying to get through the process.”  3.8.3.5 - “I didn't really have much of a queer community when I was going through my transplant. Yeah, so I feel kind of sad because I feel like if I did have one I probably would have had more support. And maybe some like tips and tricks on like what to do with people with the microaggressions at least.”  3.8.3.6 - “I had one person on a call saying that religion needs to be utilized more in the education of kidney disease and I had to bring up to him the LGBTQ community in a lot of cases has a lot of issues with religion, especially now, because it often been used as a tool to attack us so that's not where a lot of us are going to go for information about kidney disease, or kidney disease support.”  3.8.3.7 - “When I say my community, I'm really talking about the LGBTQ+ community. That's really where I identify, that's where all my friends are, where I feel supported and uplifted... What I found within my community is that people didn't have a lot of knowledge around organ transplantation. But there was a lot of compassion and support, and my community really lifted me up. In some ways this is about giving back too.”  3.8.3.8 - “I've made a few connections since starting this, with other people in other countries. So yeah, there's been a little bit of networking that's happened just to try to help change these policies.”  3.8.3.9 - “I think of our group and there was a trans man who talked about how he put the posting up that he was looking for an organ and how much support he's gotten from the community.” |
| 3.8.4 – Pediatric Considerations | 3.8.4.1 - “The thing about sexual orientation is that it can't be ignored, right? You can't hide it and I was scared if I told my care team they would discriminate against me. That added a real sense of fear growing up. Just growing up gay is hard. But growing up as a transplant recipient gay - can you imagine that? It's really difficult. I felt this like extreme low sense of self-esteem. Adding all these identities onto me… like disabled and person of color and queer on top of that… It just felt kind of overwhelming and I had such low self-esteem and then that kind of like impacted the first time I had sex because in all honesty the first time I had sex was sexual assault. I was essentially talked to like this random stranger online and then I kind of like led myself down this road where I got taken advantage of and essentially raped by a random stranger.”  3.8.4.2 - “Being a pediatric queer patient has its own caveats because I would like to just emphasize - you don't even know who you are yet and you're hit with this big thing. This tremendous amount of pain that you experienced at such a young age, and then being able to process that trauma, and then having the queer identity on top of it which is already stigmatized… So you have to learn how to exist in this world as you are at such a young age and it's really really hard. Overall, that affects us. It’s a little bit different than someone that had a transplant when they were older. We have different set of experiences. We are similar, but also the pediatric transplant person's experiences establishing their identity, establishing who they are… And identity is so important the queer experience. And identity is so important to the transplant experience. There are these things for the pediatric transplant recipient, that just might be a little bit different than someone with an adult transplant recipient experience.” |
