# Supplementary material for: Lived Experiences of Sexual and Gender Minorities in Solid Organ Transplantation: A Best-Fit Framework Synthesis and Inductive Thematic Analysis
Source: Can J Kidney Health Dis. 2025 May 29;12:20543581251331703. doi: 10.1177/20543581251331703 (PMC12126676; doi:10.1177/20543581251331703)
Supplement: sj-docx-3-cjk-10.1177_20543581251331703 – Supplemental material for Lived Experiences of Sexual and Gender Minorities in Solid Organ Transplantation: A Best-Fit Framework Synthesis and Inductive Thematic Analysis [file sj-docx-3-cjk-10.1177_20543581251331703.docx]

Appendix 3 – Theme 2 – Additional System & Structural Elements: Inclusive care

3.4.4 – Physical Spaces

Interviewees recommended the use of ambassadorial symbols (e.g., the rainbow flag) in care spaces as a simple act to enhance inclusive care through a welcoming environment that facilitates engagement with care providers. Most interviewees felt their OTDT healthcare facilities could do more to be inclusive(Appendix 3, Table 1, Quotes 3.4.4.1-3.4.4.3).

3.4.5 - Representation

Representation of SGM identities in clinical spaces facilitated inclusive care. Interviewees recognized the impact of their own visibility as SGM patients. Representation of SGM identities in medical training was felt to be critical. “The education needs to start by exposure during their medical training and programs that are designed to not just talk about (SGM identities) but expose people to (them). Panels with transgender people on them, panels with multi-cultural faces. There are different perspectives coming from the different cultures.” (Appendix 3, Table 1, Quotes 3.4.5.1-3.4.5.5).

3.4.6 – Enhanced sociodemographic variable collection

Participants said they were rarely, if ever, asked about gender identity, pronouns, used name, or sexual behaviors outside of donors who were asked which gender they had sex with during the donor risk assessment. Enhanced sociodemographic variable collection in the OTDT system was uniformly supported to enhance inclusion along with action to mitigate inequities, to ensure disclosed personal identity information is used appropriately (Appendix 3, Table 1, Quotes 3.4.6.1-3.4.6.6).

3.4.7 – Screening for HIV and Viral Hepatitis

Interviewees felt that existing policies that consider any sexual contact between men within the past 1-5 years as increasing the risk of HIV or viral hepatitis were out of keeping with actual window periods for viral detection when using nucleic-acid amplification testing (NAAT). Participants supported universal screening of potential organ and tissue donors with NAAT regardless of their sexual orientation or gender identity (Appendix 3, Table 1, Quotes 3.4.7.1-3.4.7.7).

Appendix 3, Table 1 – Representative Quotations

| Sub-Theme | Representative Quotations |
| --- | --- |
| 3.4.4 – Physical Spaces | 3.4.4.1 - “When I see a provider who has a rainbow or something it makes me feel a lot better, no matter how they identify… We're having a lot of backlash against LGBTQ identities and it is becoming even more important to be able to see that.”  3.4.4.2 - “They could probably benefit from having some kind of flag, little placard, maybe even a sticker, like this is a safe place.”  3.4.4.3 - “there's no little Pride sticker by the front desk, or little flag waving, or any of that. There's pretty much just the waiting room with a TV with videos about your program… and that's really about it.” |
| 3.4.5- Representation | 3.4.5.1 - A visibly queer (healthcare provider) would let me know that this environment is safe… if they feel safe, this environment must be safe. And I would be assured that those questions are being asked by someone who understands. I know that we can't just, you know, place queer people out there. But, just having to see in these different environments, people like me reflected is really important... I think this is another thing that a lot of people don't understand that… LGBTQ+ identities or queer identities, that we have a culture. We have a way that we speak, we have a way that we think about politics, a way that we interact. We have a way that we engage the world and so you can… you can be an ally and say really great things, but having someone who's part of your culture is really important as well, across the board.”  3.4.5.2 - “I had a septic kidney infection not too long ago and I got put on the transplant floor. There was a tonne of queer people. I had a wonderful gay male nurse. Half of my doctor team was queer and visibly queer. Like they wore rainbows, they had queer haircuts, like it was very affirming. And the whole, the whole experience felt great. My nurses, I had several queer nurses, it was just… It was really wonderful and so they would ask questions in ways that were culturally appropriate.”  3.4.5.3 - “I'd like to think that because we are gay and it was a really heartwarming story and there is a video attached to it that it took off… You know, especially like queer people around the world, it really went around the world. And maybe that gave them some hope and some glimmer of hope there, maybe some pride too and that's why it was like shared so widely.”  3.4.5.4 - “I would say in the last 10 years I've noticed a great increase in LGBT posters, materials, that those kind of things at doctors’ offices at clinics, walk in, as well as just through the hospital. I've noticed that again, the younger set of physicians are more engaged with (queer initiatives).”  3.4.5.5 - “The education needs to start by exposure during their medical training and programs that are designed to not just talk about (queer identities) but expose people to (them). Panels with transgender people on them, panels with multi-cultural faces. There are different perspectives coming from the different cultures.” |
| 3.4.6 – Enhanced sociodemographic variable collection | 3.4.6.1 - “I think one of the best ways to ask a patient about their sexual or gender identity would be… I think a questionnaire does a pretty good job. It can be uncomfortable for the provider or the patient face to face.”  3.4.6.2 - “Just having the checkboxes, I don't find that offensive. As long as there's lots of boxes for everyone to check. But there's lots of identities, all of the identities that people associate themselves with.”  3.4.6.3 - “The normalizing of collecting this information, just standardizing it, showing that we're collecting this information for everybody so that people don't feel like they're being singled out. The provider isn't bringing any assumptions into the interaction and the patient doesn't perceive that either.”  3.4.6.4 - “Also recognizing that it's not necessarily a one-time thing that you do on the intake the first time that you see a patient. Because… identity can be fluid, but also people's comfort with disclosing may change as they develop a rapport with their provider. So providing people the opportunity to update their information or to revisit these conversations I think also is important.”  3.4.6.5 - “I just don't like to think that they might use that information to discriminate and, so I guess. There might be an issue, I might check a box and think, I wonder what they're you know, gonna do with this information and how it's gonna affect how they see me and how they treat me, and if they'll discriminate against me. But you know, in a perfect world I wouldn't really care about, you know, ticking a box and on a form that says that I'm queer or that I'm gay.”  3.4.6.6 - “It is important to have face-to-face discussions about this in terms of talking about, specific concerns that people may have related to their identity. And I think opening the space, including the general discussions that patients and providers have with one another is important.” |
| 3.4.7 – Screening for HIV and Viral Hepatitis | 3.4.7.1 - “I think that those timelines are all very arbitrary and not at all tied to the accuracy of any kind of tests or the science of being able to detect infections. It's very stigmatizing for (the queer) population as well and it leads to a lot of confusion, and I think anger within a potential organ donor pool. Because they feel that stigma, and it essentially turns them off of the whole system. Having some sort of data driven timeline and removing that confusion of having different timelines for different kinds of tissues vs. organs vs. blood I think would greatly help to streamline things and remove stigma.”  3.4.7.2 - “For the NAAT test if you implement (universal screening), you take out the issue of donation restrictions in terms of months. And if that doesn't make our health care system more effective afterwards, I don't know what will. I don't know how you could not implement (routine screening) already.”  3.4.7.3 - “Universalizing these practices and making it the standard of care for everybody… if we're talking about the safety of the organ pool I think that's the best solution.”  3.4.7.4 - “I don't understand why we haven't already (implemented universal screening with NAAT) because you risk a fallout when you decide not to go with these methods and maybe trust somebody who fits a heteronormative archetype and people unfortunately in this day and age, they will lie. Many people would lie for a loved one if it meant being able to do something like donate. This would take out that risk for everybody in my opinion. Even people who fit your classic demographics. It just seems like the smart choice.”  3.4.7.5 - “I think we should use it on everybody because it's safer that way and it doesn't target specific groups regardless of behavior. I’m a social scientist and we all know that even if you ask people about their behavior, they can lie and that happens all the time too.”  3.4.7.6 - “Do we know the behavior when someone is sleeping in others beds? Does the family making the decision to donate know the behavior? No one knows what peoples’ private lives are. So to say that we know someone who's in a straight marriage doesn't have multiple sexual partners and the wife might not know or the husband may not know, is a joke. We don't know.”  3.4.7.7 - “Even when you're asking someone about their behaviors, people can be untruthful, people can lie. So you know, to me it would make sense to use this test on everyone. I don't understand unless it's just cost, you know. Money seems to be the root of a lot of evils, so is it expensive to test everybody? Probably, but would it make for a better system where you know that people are tested? So there's no question, I say, why not? Why wouldn't we do that? Use that test on everyone, it makes sense. It seems like it's a no brainer really. It would be safer for recipients and then you wouldn't have to label people increased risk or any of those sorts of things.” |
