# Supplementary material for: Lived Experiences of Sexual and Gender Minorities in Solid Organ Transplantation: A Best-Fit Framework Synthesis and Inductive Thematic Analysis
Source: Can J Kidney Health Dis. 2025 May 29;12:20543581251331703. doi: 10.1177/20543581251331703 (PMC12126676; doi:10.1177/20543581251331703)
Supplement: sj-docx-4-cjk-10.1177_20543581251331703 – Supplemental material for Lived Experiences of Sexual and Gender Minorities in Solid Organ Transplantation: A Best-Fit Framework Synthesis and Inductive Thematic Analysis [file sj-docx-4-cjk-10.1177_20543581251331703.docx]

Appendix 4 – Theme 7 - Intersecting Identities

3.9.1 – Socioeconomic Status & Geography

Socioeconomic status was relevant for transplant recipients seeking living organ donor who commonly need to provide financial support to living donors during the workup and post-transplantation care. Economic privilege, including the ability to afford comprehensive medical insurance can minimize the experience of discrimination by affording access to care for SGM people. Geographic privilege from living in socially progressive locations was identified as an important facilitator of inclusive care for SGM populations in numerous quotes (Appendix 4, Table 1, Quotes 3.9.1.1-3.9.1.2).

3.9.2 – Race, Ethnicity & Migrant Identities

In some instances, co-existing ethnic identities were advantageous for participants, especially when they were associated with a formal network. Racialization was also associated with a differential burden of preparation and performance for patients seeking care. Interviewees noted the compounding complexities that come with multiple intersecting identities. Immigrant identities were described as impacting self-advocacy and trust in the western health system (Appendix 4, Table 1, Quotes 3.9.2.1-3.9.2.6).

3.9.3 – Age

Pediatric patients occupied a unique intersection with respect to their evolving sexual orientations and their evolving identity as a medicalized transplant recipient (Appendix 4, Table 1, Quote 3.9.3.1).

3.9.4 – Ability

Interviewees had varied responses when asked if they identified with the disability community. Some transplant recipients who identified as disabled said this identity had a significant impact on the way they move through the world (Appendix 4, Table 1, Quote 3.9.4.1).

Appendix 4, Table 1- Intersections

| Sub-Theme | Representative Quotations |
| --- | --- |
| 3.9.1 – Socioeconomic Status & Geography | 3.9.1.1 - “We need to do better on policy around how we supplement the cost of organ transplant and donation. In the US, when I had my transplant done, I paid for all of my donor's expenses for her recovery period. I paid for her to get to Philly. I paid for her to stay in Philly. I paid for her during the recovery period and how that really works here is you pay the equivalent of that person's salary, plus all of their expenses that they would have in the process of donating an organ and recovering. You can't pay them for the organ in the US, but for people who don't have the resources… So we have a problem with live organ donation that we don't have enough people to do that. But it makes sense because we don't have any incentives. It's not necessarily that people don't want to donate. I had, you know, 70-something people who came forward who said they would be willing to donate to me, part of that is how do you take off time from work?”  3.9.1.2 - “My experience has been positive because I have economic privilege and geographic privilege. I'm very lucky that I am privileged that I have good insurance that pays for everything.” |
| 3.9.2 – Race, Ethnicity & Migrant Identities | 3.9.2.1 - “Being Jewish also has its own privileges. There's a great organization run by an Orthodox organization. They do a lot of networking stuff to get people kidneys. I was going through them, but then I got a cadaver donor. But they do a lot of connecting and you know, there's privilege in being able to do that because if I was Catholic, Christian, Muslim, Buddhist, whatever, they would deny me and it says so on their website.”  3.9.2.2 - “If you sound a certain way, walk a certain way, or act a certain way, you will have people saying, Oh! I don't think of you as black. It's one of the worst things you can say, but it's said quite often, just because the way that I speak, my educational background, the way I present myself, the professional avenues I walk in… I have heard ‘I don't think of you as black or one of the things that ticks me off the most is when someone says to you, ‘your diction is just amazing.’ … One of the areas that it is most notable in is the ER room. You can have 2 patients side by side, same color. And if you start talking and you're asking questions, and you're providing information… they will attend to you more.”  3.9.2.3 - “I am a black male. I am a black, gay male. And I am a gay, black, male with a transplant. And you combine all that together as long as you can find at least 1 to 2, 3 commonalities, you're doing good.”  3.9.2.4 - “When people look at me they see a small black girl. And then you also add in my identity as queer. I'm queer, I'm black and not white, and I'm a woman. So I have like everything going against me.”  3.9.2.5 - “Every experience I have is filtered through the intersection of the various different identities that I carry. I think that they all inform the way that I interact with everybody including my care team. Again, for example being an Asian American, being the being a child of immigrants that also impacts the relationship I have with my health care providers. It took me a while… there are cultural reasons why. Oftentimes it felt uncomfortable for me to speak up and advocate for myself in healthcare interactions, particularly early on when I was younger, as I was getting used to navigating care on my own. And it took some time to learn to stand up for myself and to speak up. It's hard for me to necessarily tease out like specifically like how each of those things interacts. But I know for sure that there is some interplay between all those things.”  3.9.2.6 - “Understand my background, maybe not everyone might think this way, but I am essentially the child of immigrants. I am a heart transplant recipient and I am a person of color too and in my household, there is medical mistrust, and that might seem a little weird because my life was saved by doctors, but I always feel that there's this gap between me and doctors where there's this hierarchy and that can't really be bridged. I know that doctors don't think this way. They've tried to say things like, ‘I communicate with the patient, like the patient knows that they can ask me anything’, but really there is this gap between me and the doctor and I always felt that kind of uncomfortableness there.” |
| 3.9.3 – Age | 3.9.3.1 - “Being a pediatric recipient, you're still growing up. That’s one of the differences between pediatric and adult recipients, most adult recipients are already established. They already have a career. They already have their sense of who they are. And they also have their sexual orientation figured out. Versus being a pediatric recipient, you're still figuring things out. And you're hit with this really, really big thing. This huge traumatic experience as a child. For me, it was the heart transplant. And learning how to deal with that can feel overwhelming. And so when you add on something like being gay on top of that and trying to figure out how to exist in this world, it makes it really difficult.” |
| 3.9.4 – Ability | 3.9.4.1 - “I identify as a part of the disability community based on my immunosuppression and having a transplant. I think often times we think of mobility and sensory disabilities, but chronic illness, does definitely fit into the umbrella of many shared experiences within the disability community. And even though that impact waxes and wanes and changes over time and maybe is at a minimum right now compared to other times in my life, I think the perspectives that I've gained from that lived experience and the shared experience that I have other with other disabled people informs a lot of the way that I approach the world.” |
